# Engineering MXenes: Tunable Mechanical Properties and Applications in Structural Systems

**DOI:** 10.3390/ma19102005

**Published:** 2026-05-12

**Authors:** Elijah Biggs, Amelia Bogard, Jacob Attebery, Parker Auerweck, Dakota Blaha, Subin Antony Jose, Pradeep L. Menezes

**Affiliations:** Department of Mechanical Engineering, University of Nevada-Reno, Reno, NV 89557, USA; ebiggs04@gmail.com (E.B.);

**Keywords:** MXenes, two-dimensional materials, transition metal carbides and nitrides, MAX phases, materials engineering, materials science, composite materials

## Abstract

MXenes are an emerging class of two-dimensional (2D) transition metal carbides, nitrides, and carbonitrides with a unique combination of mechanical, electrical, and thermal properties. While MXenes have been extensively studied in electrochemical and materials science contexts, their mechanical behavior and engineering relevance remain comparatively underexplored. This paper provides a mechanically focused synthesis of MXene research, connecting structure, synthesis, processing, mechanical properties, and functional performance to engineering applications. Emphasis is placed on the tunability of tensile, elastic, shear, and thermomechanical properties through controlled variation of composition, surface terminations, and defects. Comparisons with graphene are used to clarify performance trade-offs and application-specific advantages. Key challenges, including environmental stability, moisture sensitivity, durability, scalability, cost, and integration with conventional engineering materials, are critically examined alongside current mitigation strategies. Applications in structural composites, mechanical reinforcement, energy storage, electromechanical systems, and MXene-based sensors and actuators are discussed to demonstrate practical relevance. By framing MXenes as engineerable materials rather than isolated nanomaterials, this work serves as a technical reference and entry point for mechanical engineers and interdisciplinary researchers seeking to design and deploy MXenes in advanced engineering systems.

## 1. Introduction

MXenes are a class of two-dimensional (2D) transition metal carbides, nitrides, and carbonitrides first reported in 2011 by researchers at Drexel University. These materials are derived from layered MAX phase precursors and retain the hexagonal symmetry of their parent structures. The earliest MXenes synthesized were titanium carbides, and Ti-based systems remain the most extensively studied due to their relative stability and ease of fabrication [1,2,3].

MXenes originate from layered ternary carbides and nitrides known as MAX phases (M_n+1_AX_n_), where M is an early transition metal, A is an A-group element, and X is carbon and/or nitrogen. At the time of MXene discovery, approximately 70 MAX phases had been identified; that number has since more than doubled, with dozens successfully converted into MXenes. This expanding compositional space has enabled systematic exploration of structure–property relationships across a growing family of 2D materials [2,4].

MXenes combine high electrical conductivity, tunable surface chemistry, and mechanical robustness comparable to other high-performance 2D materials such as Graphene. Their surfaces are terminated by functional groups introduced during synthesis, allowing control over conductivity, surface energy, hydrophilicity, and oxidation behavior. Through compositional selection and termination engineering, properties can be tailored to specific application requirements, including structural reinforcement, energy storage, sensing, and electromechanical integration [1,5].

Despite rapid progress, challenges remain. Many MXenes exhibit sensitivity to moisture and oxidation, which can compromise long-term mechanical and electronic stability. Scalable, cost-effective production and integration with conventional engineering materials continue to present practical barriers. Furthermore, only a fraction of theoretically predicted MXene compositions have been experimentally realized, and fewer still have been implemented in application-ready systems [1,2,6].

This paper presents a mechanically oriented synthesis of current MXene research, examining structure and composition, synthesis and processing strategies, mechanical and functional properties, and application-driven design considerations. Emphasis is placed on tunability through surface terminations and defect engineering, as well as the challenges that must be addressed to transition MXenes from laboratory materials to deployable engineering systems.

## 2. Structure and Composition of MXenes

### 2.1. General Formula and Origin

MXenes are a family of crystalline, atomically thin materials belonging to the broader class of 2D solids. Individual MXene layers are typically only a few atoms thick and can exist as isolated sheets or be assembled into stacked, multilayered architectures, enabling tunable properties across multiple length scales [7]. Their composition and atomic arrangement govern both interlayer interactions and interfacial behavior with surrounding materials, directly influencing mechanical, electrical, thermal, and chemical performance [8].

The general composition of MXenes is described by the formula M_n+1_X_n_T_x_, where M is an early transition metal (commonly from groups 3–6), X represents carbon and/or nitrogen, and T_x_ denotes surface termination species. In the most standard form, MXenes possess a middle layer composed of the X-element, between an upper layer and a lower layer composed of the transition metal. Structurally, X atoms occupy octahedral sites coordinated by layers of M atoms, forming a layered carbide or nitride framework. Surface terminations bond to the outermost M layers and are an intrinsic feature of most experimentally realized MXenes [7,9,10].

MXenes are derived from layered MAX phases, which use the formula M_n+1_AX_n_, where A is an element from groups 13–15 of the periodic table. Selective chemical removal of the A layer, performed through etching, transforms the MAX precursor into a MXene by exfoliating the layered structure and introducing surface terminations. As a result, the A element is largely eliminated, while the transition metal, a carbon/nitrogen framework, is retained and functionalized [8,11].

Early MXene research focused on a relatively small number of experimentally accessible compositions (fewer than 70 known phases), but the number of reported and predicted MXene structures has doubled with advances in synthesis and computational screening [8]. MXenes share several structural features with graphene, including crystallinity, atomic-scale thickness, and in-plane hexagonal symmetry. This resemblance motivated the “-ene” suffix in their nomenclature, reflecting their two-dimensional nature rather than direct chemical similarity.

Most commonly, MXenes consist of a central X atomic layer located between two M layers, with surface terminations bonded to the exterior metal planes. This ordered sequence provides the structural foundation for the diverse range of MXene compositions and properties explored in current research [8,11].

### 2.2. Compositional Differences of MXenes and MAX Phases

MAX phases incorporate an additional *A*-site element, most often an A-group metal such as aluminum or silicone which plays a critical role in stabilizing the layered precursor structure [8,12]. In some less common cases, elements from the B-group of the periodic table (e.g., iron or copper) may occupy analogous structural roles [12]. During conversion to MXenes, this A (or B) component is typically removed, resulting in a chemically and structurally distinct material despite the shared transition-metal-carbon/nitrogen backbone.

Representative examples highlight this distinction. For instance, Ti_3_AlC_2_ is a well-known MAX phase containing aluminum as the *A* element, whereas its corresponding MXene derivatives (e.g., Ti_3_C_2_T_x_ or Ti_3_C_2_Cl_2_) lack the aluminum layer and instead possess surface terminations introduced during synthesis [10]. These compositional changes fundamentally alter bonding, electronic structure, and environmental stability.

MXenes are frequently processed and utilized in powder, flake, or film form rather than as bulk monoliths. This preference reflects both their atomically thin morphology and the relative fragility of individual layers under mechanical or chemical stress. Many MXenes are electrically conductive and strongly hydrophilic, which enhances dispersion in aqueous environments but also increases susceptibility to oxidation and degradation, particularly under humid or oxygen-rich conditions [9].

The choice of transition metal, as well as the specific synthesis pathway, strongly influences the resulting atomic ordering, defect density, and termination chemistry of MXenes [12]. Consequently, the structure of a MXene cannot be fully decoupled from its production method. Because a valid MAX-phase precursor is generally required to obtain a corresponding MXene, compositional design is constrained by both thermodynamic stability and synthetic feasibility [13,14]. Variations in the precursor structure can affect atomic positions within the resulting MXene lattice, including the distribution of *X* atoms and the local coordination of transition metals [14]. These compositional and structural dependencies underscore the close coupling between chemistry, processing, and final material properties in MXenes.

### 2.3. Types of MXenes

One of the defining strengths of MXenes is the flexibility of their composition and structure, which allows a wide range of properties to be accessed through controlled modification [8]. The simplest and most widely studied class consists of single-transition-metal MXenes, which follow the standard M_n+1_X_n_T_x_ structure described previously.

More complex architectures are achieved by introducing additional transition metals. Double-transition-metal MXenes incorporate two distinct M elements while maintaining an ordered atomic arrangement. These materials are commonly categorized as in-plane or out-of-plane double-transition-metal MXenes [8,12]. In in-plane structures, both transition metals are distributed within the same atomic layer, whereas in out-of-plane structures, the second transition metal forms distinct layers alternating with those of the primary metal [7,12].

A further level of compositional variation is found in solid-solution MXenes, typically described by the formula (M, M′) X T_x_. In these materials, multiple transition metals are randomly distributed within the same lattice sites rather than occupying ordered positions [9,12]. Solid-solution MXenes also allow mixed *X* components, enabling the simultaneous incorporation of carbon and nitrogen within a single structure.

More recently, high-entropy MXenes have been proposed as an extension of this concept, incorporating three or more transition metals into a single lattice [12]. Although experimental examples remain limited, this approach has the potential to significantly expand the accessible compositional space and enable fine-tuning of properties. The specific MXene type that forms depends not only on elemental selection but also on synthesis conditions, which impose practical constraints on atomic ordering and phase stability. Table 1 summarizes the compositional distinctions among the primary MXene structural classes discussed above.

### 2.4. Role and Impact of Surface Terminations

Surface terminations are a defining feature of experimentally synthesized MXenes and play a central role in determining their properties. A single MXene structure can support several termination species simultaneously, and as early as 2015 it was demonstrated that individual MXenes may host up to three different surface terminations on their outer metal layers [8].

These termination groups bond to the outermost *M* atoms and strongly influence electrical conductivity, chemical reactivity, thermodynamic stability, and interfacial behavior [15]. The nature of the surface terminations is closely tied to the synthesis method, as termination species are typically introduced during chemical etching and post-processing steps [16]. Advances in controlled synthesis have enabled partial regulation of termination chemistry, allowing greater control over MXene behavior and performance.

**Figure 1:**
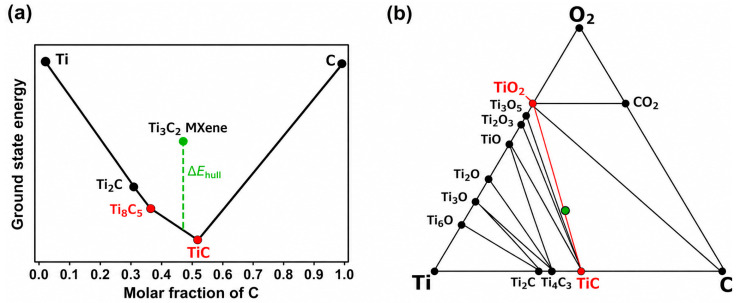
Computation of the Energy Above Energy Convex Hull for (**a**) Ti_3_C_2_ and (**b**) Ti_3_C_2_O_2_ MXenes using binary Ti–C and ternary Ti–C–O convex hull phase diagrams, respectively. Reproduced from [17], Open access.

Surface terminations also have a critical impact on MXene stability. Many MXenes are classified as thermodynamically unstable or metastable, with stability strongly dependent on the type and distribution of termination species [16,17]. MXenes, which are terminated with halogen elements from group 17, such as fluorine or chlorine, are among the most thermodynamically stable. Even so, fewer than 30% of theoretically possible MXene compositions are predicted to be stable under equilibrium conditions [17]. This limitation underscores the importance of termination chemistry in determining which MXenes are viable for practical use. Ground state energy is also affected by MXene composition, as seen in Figure 1.

Due to their layered crystalline structure and frequent use in stacked or powder forms, MXenes are generally considered porous materials. This porosity enables interactions with other materials and molecules, facilitating the design of composite and hierarchical structures. For example, MXene frameworks have been used to anchor micro- and nanoscale structures, such as microtubes, and to form folded or spherical architectures through controlled assembly processes [18,19]. While individual MXene layers are rigid at the atomic scale, larger-scale assemblies exhibit a high degree of structural tunability.

### 2.5. Limitations and Outlook

Despite the broad compositional flexibility of MXenes, current research is heavily concentrated on titanium-based systems. As of 2023, approximately 70% of reported MXene studies focus on Ti-based compositions [15]. As a result, the remaining viable transition metals—including scandium, vanadium, yttrium, zirconium, niobium, molybdenum, hafnium, tantalum, and tungsten—are comparatively underexplored [2].

**Figure 2:**
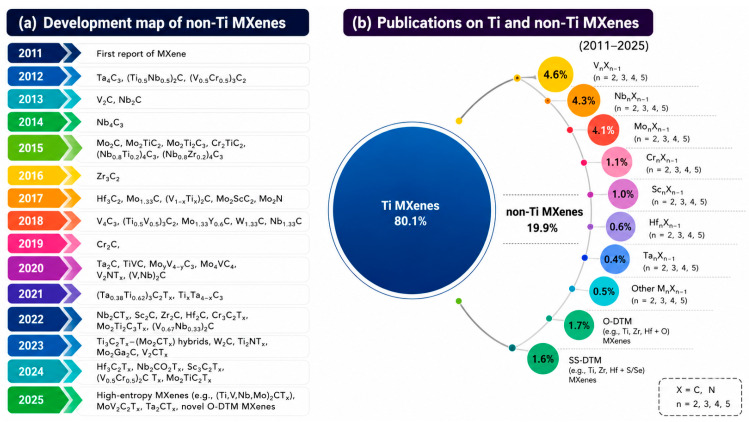
Evolution of non-Ti MXenes and comparative publication trends of Ti and non-Ti MXenes from 2011 to 2025, with (**a**) showing representative non-Ti MXene developments, and (**b**) the approximate bibliometric distribution of Ti- and non-Ti-based MXene research. Data adapted from bibliometric trends and representative MXene literature.

Computational and experimental studies suggest that additional elements, including certain lanthanides and actinides, could potentially serve as *M* components in MXenes, further expanding the theoretical compositional space [14,17]. However, this expansion also disperses research efforts and compounds existing challenges related to synthesis feasibility and material stability. Figure 2 depicts the fraction of studies related to Ti-based MXenes compared to non-Ti MXenes as of 2025.

Structural and compositional constraints remain major limiting factors in the advancement of MXenes. Many compositions suffer from limited thermodynamic stability, and their atomically thin, rigid microstructures can be mechanically fragile when scaled to larger assemblies. These characteristics complicate the fabrication of large, free-standing MXene sheets or load-bearing structures [20]. Addressing these limitations through improved compositional design, termination control, and structural engineering is essential for expanding the range of viable MXene applications.

## 3. Synthesis and Processing of MXenes

### 3.1. Selective Etching of MAX Phases

The synthesis of MXenes was first reported in 2011 and has expanded substantially with respect to both the number of available compositions and the diversity of processing routes. As of the late 2010s, approximately 30 MXenes had been experimentally synthesized and characterized in detail, while many additional compositions remained theoretically predicted but not yet realized experimentally [21,22].

MXene synthesis generally begins with the preparation of a suitable MAX-phase precursor, which serves as the parent layered material. [21,23] Several classes of MAX phases exist, including conventional MAX phases as well as variants containing multiple A elements or alternative atomic substitutions. Regardless of the specific precursor, the defining step in MXene synthesis is the selective removal of the A-layer, while preserving the strong covalent bonding between the transition metal (M) and carbon or nitrogen (X) [23,24]. This etching process must be carefully controlled to ensure that the resulting M-X layers retain their two-dimensional structure rather than collapsing or transforming into non-layered phases. Successful etching exposes the layered carbide or nitride framework and enables subsequent exfoliation into few-layer or single-layer MXenes, forming the basis for most downstream processing routes.

### 3.2. Top-Down and Bottom-Up Approaches

MXene synthesis routes are commonly classified as either top-down or bottom-up approaches, with top-down methods currently dominating experimental and industrial research. In top-down synthesis, a layered precursor, most often a MAX phase, is selectively etched and exfoliated to obtain two-dimensional MXene sheets. In contrast, bottom-up approaches aim to directly grow MXene structures from atomic or molecular precursors, rather than removing layers from an existing bulk material [25].

Historically, the most widely used top-down methods relied on aqueous fluoride-containing solutions, such as hydrofluoric acid or in situ HF-generating systems [26]. While effective, these methods raise significant safety and environmental concerns, motivating the development of fluoride-free etching techniques [26,27]. Prominent fluoride-free approaches include HCl-based electrochemical etching, Lewis-acid molten-salt etching, and halogen-based etching [27].

In HCl electrochemical etching, an applied potential drives the selective removal of the A-layer within an electrochemical cell. While effective, excessive etching can result in carbon-derived carbide overlayers that partially obscure the MXene surface [27]. Lewis-acid molten-salt etching employs molten salts at elevated temperatures (≈750 °C) to chemically extract the A element through controlled cation exchange, enabling the synthesis of MXenes inaccessible by conventional HF routes [27]. Halogen-based etching methods exploit the high reactivity of halogens such as Cl_2_, Br_2_, and I_2_ to selectively remove A layers without fluorides, though these methods require strict safety controls [27].

Additional fluoride-free, top-down techniques include microwave synthesis (MW), molten-salt synthesis (MS), solid-state reaction (SSR), and pressureless sintering (PLS) [26,27]. Microwave synthesis enables rapid and localized heating, achieving high temperatures with short dwell times, though sample sizes remain limited [28]. Molten-salt synthesis and solid-state reaction routes rely on high-temperature diffusion and reaction kinetics, often requiring protective atmospheres to prevent oxidation. Pressureless sintering favors sample homogeneity but involves long processing times and low heating rates [28].

Closely related high-temperature consolidation methods, hot pressing (HP), hot isostatic pressing (HIP), and spark plasma sintering (SPS), apply pressure-assisted densification during synthesis. Among these, SPS offers particularly rapid heating rates and short dwell times, though scalability is limited by mold requirements [28].

Quantitative comparisons across synthesis methods highlight significant differences in flake size, defect density, and scalability. For example, HF-based and electrochemical etching methods typically produce flakes on the order of 1–10 μm with moderate defect densities, while molten-salt approaches can yield larger multilayer structures (>10 μm) but with increased interlayer bonding that complicates delamination. In contrast, bottom-up methods such as PVD produce highly uniform, low-defect films with nanometer-scale thickness control, though lateral dimensions are constrained by deposition geometry and throughput is limited. Yield and scalability are highest for liquid-phase and molten-salt top-down methods, which are more readily adapted to batch processing, whereas bottom-up approaches remain restricted to thin-film applications and small-scale production [29,30].

Bottom-up synthesis methods are less common but offer advantages in producing high-quality, large-area MXenes. Physical vapor deposition (PVD) is the most established bottom-up approach and includes cathodic arc deposition, pulsed laser deposition, and magnetron sputtering, the latter being most widely used [28]. These techniques enable the growth of dense, high-purity MXene films at moderate temperatures but require line-of-sight deposition and complex equipment. Table 2 compares the advantages and limitations of representative top-down and bottom-up MXene synthesis methods, highlighting trade-offs between scalability, purity, processing time, and safety.

### 3.3. Challenges in Synthesis and Scalability

Despite significant progress, MXene synthesis faces several persistent challenges, particularly in large-scale production. Top-down liquid exfoliation methods often require surfactants, organic solvents, or polymer-based stabilizers to achieve high concentrations of dispersed MXene flakes, complicating processing and post-treatment steps [26].

**Figure 3:**
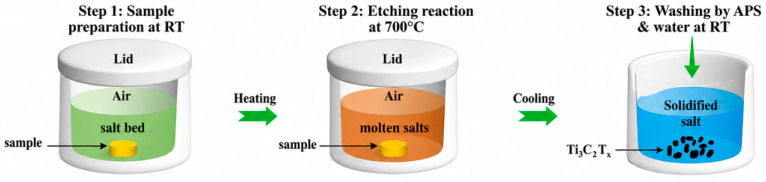
Molten Salt-shielded Synthesis of MXenes, a Top-Down Synthesis Method. Reproduced from [25], Open access.

Scaling top-down synthesis introduces additional issues, including defect formation and uncontrolled surface termination chemistry arising from aggressive etchants such as HF, LiF, or HCl [25]. One such top-down method, molten salt-shielded synthesis, is shown below in Figure 3. Bottom-up methods, on the other hand, are much better suited to high-quality, large-area 2D materials. This is due to the bottom-up method of growing them, allowing for the materials to be more flexible [25]. Mechanical agitation methods, such as sonication, can further reduce flake size and introduce structural defects, negatively impacting material performance.

Bottom-up methods, while capable of producing high-quality, low-defect, and compositionally controlled MXenes, are generally limited in throughput and scalability due to equipment constraints and line-of-sight deposition requirements. As a result, they are more suitable for thin-film and microelectronic applications rather than bulk material production. In contrast, top-down methods, particularly liquid-phase and molten-salt etching, offer higher material yield and batch scalability, making them more viable for large-scale engineering applications, despite higher defect densities and less precise control over surface chemistry [25]. Although quantitative comparisons of yield and defect density remain limited across studies, consistent trends indicate that liquid-phase and molten-salt etching methods provide higher scalability at the expense of increased defect density, whereas bottom-up approaches yield higher-quality materials but are constrained by low throughput and equipment limitations. As a result, a trade-off persists between material quality and production throughput. Addressing these challenges through improved etching control, alternative synthesis pathways, and hybrid processing strategies remains essential for the broader adoption of MXenes in engineering applications.

## 4. Mechanical Properties of MXenes

### 4.1. Strength, Toughness, and Flexibility

The mechanical behavior of MXenes encompasses several interrelated properties, including tensile strength, elastic stiffness, shear strength, and thermomechanical stability. These properties are strongly influenced by chemical composition, surface termination chemistry, layer thickness, and the presence of defects. As a result, MXenes exhibit a high degree of mechanical tunability, enabling targeted optimization for specific applications through compositional and surface engineering.

Many MXenes demonstrate high in-plane tensile stiffness, with reported values ranging from approximately 81.7 N/m to 561 N/m, determined using high-throughput computations. This depends on composition and thickness [37]. This was computed for several layer numbers, and tensile stiffness increased with layer number. Tensile stiffness is governed by bond strength within the M-X layers, the number of layers present, and surface termination chemistry, with some terminations producing stiffness enhancements approaching 100% relative to unfunctionalized surfaces [37]. These effects highlight the sensitivity of MXene mechanical behavior to atomic-scale modifications.

**Figure 4:**
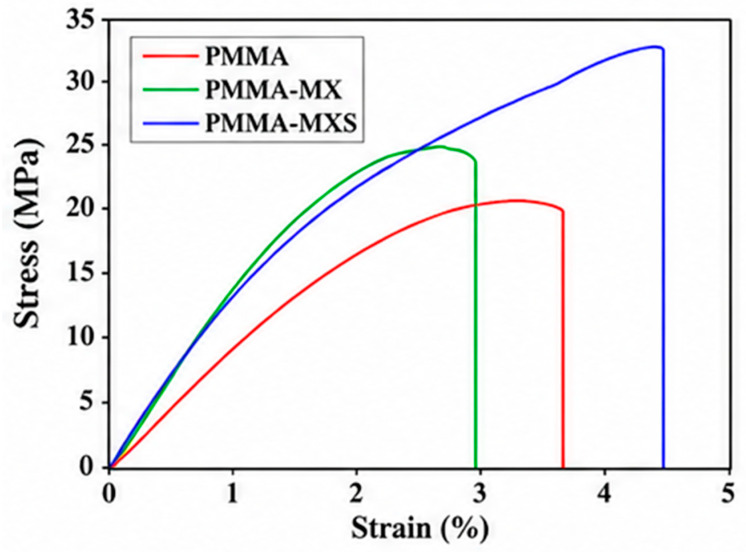
Stress–strain behavior of PMMA, PMMA-MXene, and PMMA-functionalized MXene composites. Reproduced from [38], Open access.

The reinforcing capability of MXenes is further demonstrated in polymer composites. For example, when polymethyl methacrylate (PMMA) is reinforced with 1 wt% MXenes using stable water dispersion, the resulting composites exhibit a 37% increase in tensile strength relative to neat PMMA, along with 22.1% and 27.6% increases in Young’s modulus, respectively [38]. The MXene was functionalized with MEMO silane in this case, which improves dispersion uniformity and interfacial bonding within the polymer matrix. The reported improvements are based on experimentally measured averages, though variability depends on dispersion quality and processing conditions. The corresponding stress–strain behavior is shown in Figure 4, illustrating enhanced stiffness and load-bearing capacity without catastrophic loss of ductility.

**Figure 5:**
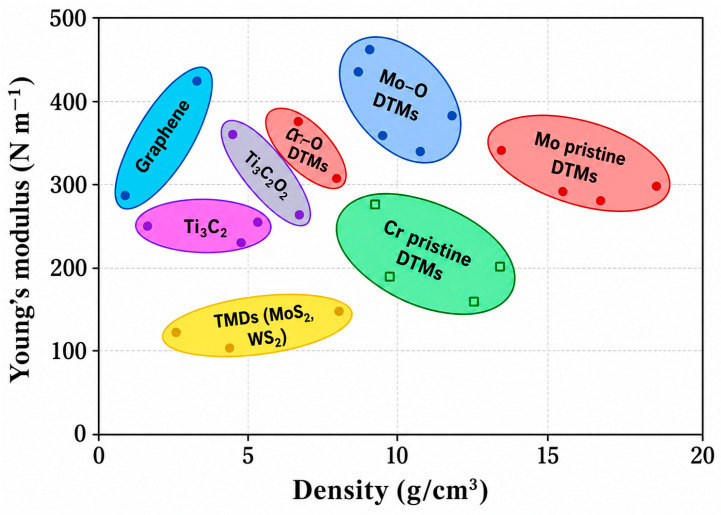
Ashby map of Young’s modulus vs. density for MXenes and Other Materials. Reproduced with permission from [39].

More specifically, the increased slope in the elastic region for PMMA-MX and PMMA-MXS indicates improved load transfer efficiency between the polymer matrix and MXene fillers. This enhancement is attributed to strong interfacial bonding, particularly in the functionalized system (PMMA-MXS), where silane coupling promotes chemical adhesion and reduces interfacial slippage. The higher ultimate stress and extended strain-to-failure observed in PMMA-MXS suggest that crack propagation is delayed through effective stress redistribution and possible crack-bridging mechanisms. In contrast, the abrupt failure observed in PMMA-MX indicates that while stiffness is improved, insufficient interfacial bonding leads to premature debonding and limited energy dissipation. These differences highlight the critical role of interfacial engineering in balancing stiffness, strength, and toughness in MXene-reinforced composites [38]. Figure 5 subsequently shows a comparison of Young’s modulus vs. density for MXenes and other materials.

Surface terminations also influence optical and mechanical responses. In PMMA-MXene systems, termination chemistry alters optical transmittance by modifying electronic structure, as evidenced by the disappearance of specific transmittance peaks [38]. Beyond passive reinforcement, hydrogen bonding between MXene sheets has been shown to significantly enhance tensile strength and toughness while enabling self-healing after mechanical damage [40]. These hydrogen-bonded MXene assemblies maintain flexibility and exhibit resistance to complex environmental loading.

The elastic response of individual MXene nanosheets further reflects their mechanical robustness. Ti_3_C_2_T_x_ nanosheets exhibit reported elastic stiffness values as high as 947 N/m, with elastic strain limits of approximately 3.2% [41]. These data were collected using nanomechanical push-to-pull equipment under a scanning electron microscope. While edge defects reduce stiffness and strain tolerance, their impact diminishes with increasing layer thickness, indicating a thickness-dependent defect sensitivity.

**Figure 6:**
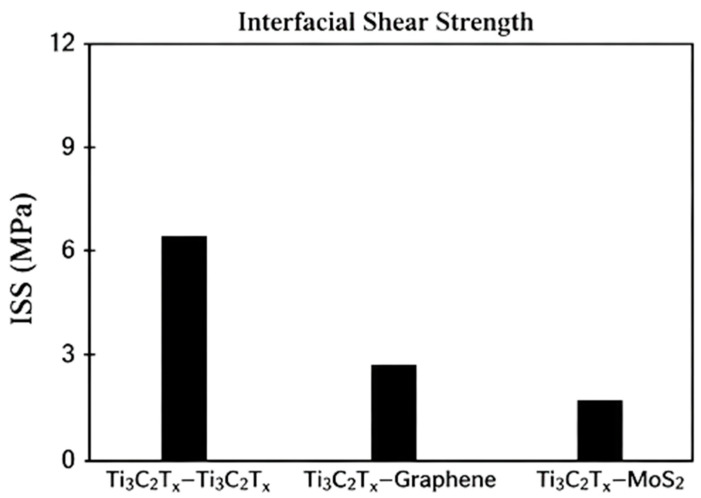
Interfacial shear strength of selected MXene and hybrid composites. Data extracted from [42], Open access.

Shear properties, particularly interfacial shear strength, are critical for layered and composite MXene systems. Comparative studies of Ti_3_C_2_T_x_-Ti_3_C_2_T_x_, Ti_3_C_2_T_x_-graphene, and Ti_3_C_2_T_x_-MoS_2_ interfaces show that MXene-MXene interfaces exhibit the highest interfacial shear strength [42]. These results, summarized in Figure 6, highlight the strong interlayer interactions achievable within MXene-based architectures.

This trend can be attributed to the combined effects of electrostatic interactions, surface functional groups, and lattice compatibility between adjacent MXene layers, which enhance interfacial adhesion and resistance to shear-induced sliding. In contrast, heterointerfaces such as MXene-graphene and MXene-MoS_2_ exhibit reduced shear strength due to weaker van der Waals interactions and lattice mismatch, which limit load transfer across the interface. As a result, failure in these systems is more likely to occur through interfacial sliding rather than cohesive fracture. These distinctions are critical for composite design, where maximizing interfacial shear strength directly improves stress transfer efficiency and overall mechanical performance [42].

Surface termination chemistry further modulates shear strength. Among common terminations (-O, -F, -OH, -Cl, -H), -H terminations yield the highest interfacial shear strength, while -F, -OH, -O, and -Cl generally result in lower values [42]. Temperature- and pressure-dependent studies reveal that different terminations are optimal under different operating conditions: -OH terminations perform best under milder conditions, whereas -O terminations are favored at elevated temperatures and pressures [43]. This behavior enables mechanical performance to be tailored for specific service environments through termination control.

Thermomechanical behavior in MXenes is closely linked to termination chemistry. For Ti_3_C_2_T_x_ MXenes, -O terminations produce the highest in-plane elastic modulus and improved thermodynamic stability, attributed to enhanced charge transfer between inner and outer surface bonds [44]. These thermodynamic effects are coupled to electronic behavior, as summarized for Ti_3_C_2_T_x_ electrodes in Table 3, which reports capacitance retention across various electrolytes and cycling conditions.

Temperature-dependent studies further indicate that MXene mechanical properties degrade at elevated temperatures. For example, the calculated Young’s modulus and tensile stiffness decrease as temperature increases from 0 K to 1000 K, consistent with bond softening at high thermal energies [45]. Comparisons between Ti_3_C_2_O_2_ and Ti_2_Co_2_ also show higher thermal conductivity in Ti_3_C_2_O_2_, in agreement with broader trends reported in the literature [45].

### 4.2. Comparison with Graphene

The advantages of MXenes are often clarified through comparison with graphene, another widely studied 2D material. Both materials share desirable physicochemical characteristics, including high electrical conductivity, large surface area, and tunable electronic properties. However, MXenes possess additional features, most notably hydrophilicity and chemically active surface terminations, that distinguish them from graphene and expand their functional versatility [5].

Surface mapping studies of functionalized MXene and graphene nanosheets indicate that forming stable monolayers at the air-water interface is challenging for both materials. Monolayer formation becomes feasible when amphiphilic molecules are introduced or when the subphase chemistry is modified. Under these conditions, MXenes exhibit enhanced interfacial adhesion due to their inherent surface roughness and termination chemistry [46].

Mechanical comparisons reveal that graphene generally exhibits higher hardness and elastic modulus than MXenes. Nanoindentation measurements show that for thin samples, hardness values are approximately 89 GPa for MXenes and 100 GPa for graphene, while thicker (~10-layer) samples show reduced hardness of 7.5 GPa and 9.8 GPa, respectively [47]. Corresponding elastic modulus values, summarized in Table 4, confirm that MXenes are mechanically softer than graphene but remain structurally robust.

The properties reported in Table 4 are compiled from experimentally reported literature corresponding to the closest comparable configurations for each material. For graphene, MoS_2_, and h-BN, values primarily reflect intrinsic or suspended monolayer measurements. In contrast, Ti_3_C_2_T_x_ MXene values are derived from experimentally synthesized single flakes or thin films that include surface terminations and structural variability. Measurement techniques include AFM nanoindentation (mechanical properties), Raman thermometry and optothermal methods (thermal conductivity, and field-effect transistor (FET) characterization (electrical conductivity). Due to differences in sample geometry, measurement conditions, and intrinsic material structure, reported values represent benchmark ranges rather than strictly equivalent intrinsic constants. These distinctions should be considered when making cross-material comparisons.

Direct one-to-one comparison between MXenes and classical 2D materials remains inherently limited by differences in synthesis routes, surface chemistry, and measurement methodologies; therefore, values are selected to reflect the closest experimentally relevant conditions rather than strictly identical testing configurations.

Despite this difference, MXenes often outperform graphene in composite reinforcement applications. When used as fillers in E-glass fiber composites, graphene increases flexural strength by approximately 30% and tensile strength by 12%, whereas MXenes increase flexural strength by 45% and tensile strength by 10% [69]. These data were collected by examining electron microscope scans. Additionally, MXene-reinforced composites exhibit half the water absorption of graphene-reinforced counterparts, indicating superior environmental resistance [33].

Functional distinctions also arise in application-specific contexts. In energy storage, graphene benefits from high carrier mobility and thermal stability, while MXenes offer advantages in thermal conductivity, electromagnetic absorption, and surface-driven charge storage [70]. In gas-sensing applications, graphene typically relies on resistance changes, whereas MXenes enable chemically selective sensing through surface reactivity with target gases such as NH_3_, NO_2_, and H_2_S, allowing greater application-specific customization [71].

In addition to MXenes and graphene, other emerging 2D materials and hybrid architectures have demonstrated notable mechanical and thermomechanical behavior. For instance, Janus transition metal dichalcogenide (TMD) heterostructures such as MoSSe–WX_2_ (X = S, Se) exhibit unique intrinsic wrinkled superlattice configurations. They contribute to enhanced flexibility and mechanical adaptability due to built-in asymmetry and interfacial strain modulation [72,73]. These wrinkle interfaces have been shown to influence thermal transport characteristics, highlighting the coupled nature of mechanical deformation and phonon scattering mechanisms in such systems. Furthermore, strain engineering in TMDs such as WS_2_ enables tunable mechanical properties, where applied strain and defect states significantly modify elastic modulus and deformation response. In addition, hybrid systems such as Fe_2_O_3_/graphene composites demonstrate improved structural stability and resistance to degradation, primarily due to the synergistic interaction between graphene’s high mechanical strength and the active material framework. Incorporating these materials into the broader context provides a more comprehensive framework for comparing MXenes with other 2D systems and underscores the importance of structure–property relationships in determining their engineering applicability [74,75].

### 4.3. Effects of Defects and Terminations

Environmental degradation remains a key limitation for MXenes, particularly under humid or oxidative conditions. Titanium vacancies (V_Ti_) act as active sites for the protonation of subsurface carbon atoms, weakening local bonding and accelerating the release of Ti atoms. This effect is most pronounced in -OH-terminated MXenes, which exhibit strong termination bonding and enhanced reactivity [68]. These defect-mediated reactions also serve as primary initiation pathways for oxidation and hydrolytic degradation, as discussed in Section 4.4.

**Figure 7:**
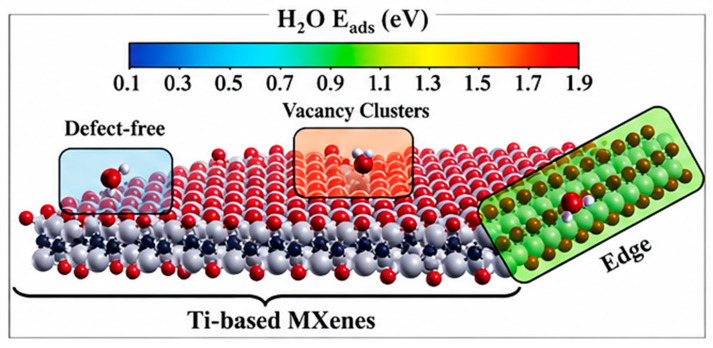
Water adsorption energies at defect and termination sites on MXene. Reproduced from [68], Open access.

Water adsorption is strongly dependent on defect type and location, with increasing adsorption observed at isolated termination vacancies, edge sites, and clustered defect regions, respectively. These trends are illustrated in Figure 7, which shows H_2_O bond energies at representative MXene sites [68]. Defect stabilization can be achieved through adsorption of metal cations such as Li^+^, Na^+^, K^+^, and Mg^2+^, which reduce water access to reactive sites and mitigate degradation. However, the long-term effectiveness of such stabilization strategies under ambient conditions remains limited due to continued oxygen diffusion and progressive oxidation [76].

First-principles studies examining vacancies in MXenes composed of Ti, Zr, Hf, V, Nb, Ta, Cr, Mo, and W indicate that vacancies generally induce outward relaxation of neighboring atoms due to reduced electronic screening and strengthened remaining bonds. Oxygen vacancies exhibit particularly high formation energies, while X-site vacancies have comparatively lower energetic penalties [77].

Defects can also be exploited to enhance functional performance. In V-based MXenes such as V_1−x_Cr_x2_AlC, moderate concentrations of point vacancies improve capacitance and charge-transfer rates, whereas excessive defect densities degrade electrochemical performance [78]. This behavior enables controlled tuning of conductivity and storage performance through defect engineering.

Targeted defect introduction can further enhance bond strength. Defects involving M-C interactions increase bonding strength, while M-Ga defects weaken it, independent of Cr concentration. Additionally, Mo-C and Mo-Ga interactions are stronger than their Cr-based counterparts, providing another route for compositional optimization [79]. Mechanical enhancement via defect engineering has also been demonstrated experimentally: introducing surface defects in Ti_3_C_2_T_x_ MXenes through sequential H_2_O_2_ and HCl treatment increases compressive strength by 36.1% and failure strain by 38% [80]. These improvements, however, must be balanced against the increased susceptibility of defect-rich structures to environmental degradation over time.

### 4.4. Environmental Degradation Mechanisms and Kinetics

MXenes, particularly Ti_3_C_2_T_x_, are susceptible to oxidation and hydrolytic degradation under ambient and aqueous conditions, which can significantly alter their mechanical and functional performance. Degradation is primarily initiated through interactions with dissolved oxygen and water, leading to the progressive formation of titanium dioxide (TiO_2_) and amorphous carbon phases. This process is accelerated by elevated temperature, light exposure, and the presence of defects or edge sites, which act as reactive centers [81,82].

Quantitative studies have shown that Ti_3_C_2_T_x_ colloidal suspensions stored in water at room temperature can exhibit measurable oxidation within days, with substantial structural degradation occurring over 1–2 weeks, depending on flake size and storage conditions. Smaller flakes and higher defect densities correlate with faster degradation rates due to increased surface area and reactivity [82]. In contrast, MXene films stored in inert or dry environments demonstrate improved stability, with degradation timescales extending to months under controlled conditions.

For example, controlled experiments reported by the American Chemical Society indicate that oxygenated surface terminations (-O, -OH) facilitate further oxidation pathways, while fluorine terminations (-F) can moderately retard degradation. Additional kinetic analyses suggest that oxidation follows a diffusion-limited process in multilayer films but is reaction-limited in dispersed systems [83].

These degradation pathways directly impact mechanical integrity. Oxidation-induced embrittlement, reduction in interlayer cohesion, and the formation of brittle oxide inclusions lead to decreased tensile strength and reduced flexibility over time. This is particularly critical for load-bearing or cyclic mechanical applications, where long-term reliability is required [76].

## 5. Functional Properties Relevant to Mechanical Engineers

Beyond their mechanical attributes, MXenes possess several functional properties of direct relevance to mechanical engineering systems. Their thermal conductivity and stability, barrier performance against gases and moisture, tribological behavior, and electrical and electrochemical characteristics [84], together with the mechanical and chemical properties discussed above, allow MXenes to serve multiple functions within integrated components and systems.

### 5.1. Thermal Conductivity and Stability

MXenes demonstrate promising behavior in terms of thermal transport and can be tailored for thermal management applications. Some MXenes have been studied as thermoelectric materials (which convert heat to electricity and vice versa). The performance of a thermoelectric is measured by the figure of merit ZT = S^2^σT/κ (where S is Seebeck coefficient, σ electrical conductivity, and κ thermal conductivity) [85]. MXenes often exhibit a favorable balance: their electrical conductivity can be high (metallic-like), and their lattice thermal conductivity is relatively low due to atomic disorder and heavy atomic masses, which is good for thermoelectric efficiency [86]. For example, certain MXenes composites have shown ZT values comparable to bismuth telluride at elevated temperatures, owing to their “ceramic-like” nature combined with tunable carrier concentration [86].

From a more general thermal management perspective (like cooling of electronics), MXenes are notable for strongly anisotropic thermal conductivity [87]. Within the plane of a MXene sheet, heat can conduct well through the covalently bonded metal-carbon lattice, but through the thickness (from one side of a sheet to the other, or across a stack of sheets), the weak van der Waals contacts and any interlayer gaps result in much lower thermal conductivity. This anisotropy is advantageous: a film or coating of MXene can spread heat laterally (due to high in-plane κ) but also serve as a thermal barrier in the perpendicular direction (due to low through-plane κ) [88,89]. Representative studies on Ti_3_C_2_T_x_ films report increasing in-plane thermal conductivity with temperature, reaching values on the order of a few W·m^−1^·K^−1^ near room temperature [90]. This property holds crucial for thermal management in devices like solar cells and electronics where it can draw heat along a surface (cooling a hotspot on a chip) while insulating what’s beneath from that heat. To illustrate the practical impact of anisotropic thermal transport. The Ti_3_C_2_T_x_ layer promotes lateral heat spreading while simultaneously reducing outward infrared emission, resulting in a visibly cooler surface compared to the uncoated regions.

The thermal stability of MXenes is another key characteristic of MXenes. Some (especially with larger transition metals like Zr or Hf) are remarkably stable: Zr_3_C_2_T_x_ has been shown to remain intact up to ~1000 °C in vacuum [91]. In air or oxygen-containing environments, most MXenes will oxidize at much lower temperatures (several hundred °C, depending on termination and environment) [81]. Thus, for high-temperature applications, an inert environment or protective coatings are needed. The stability also correlates with composition: Ti-based MXenes tend to oxidize around 150–300 °C in air (forming TiO_2_), whereas ones like Nb-based might be slightly more stable or decompose differently [81]. When using MXene as a thermally conductive additive in, e.g., a polymer composite, one must ensure the composite’s processing temperature (~200 °C for some polymers) does not cause the MXene to degrade [92]. Encouragingly, MXene/polymer composites have been made and tested for thermal conductivity improvements without issues at those processing temperatures.

Another aspect is thermal interface materials (TIMs), which are used between two surfaces to improve heat flow. MXenes’ ability to conform to surfaces (as soft, thin films or pasted as fillers) and conduct heat could be harnessed in TIMs for electronics. One study used MXene flakes mixed into a polymer to create a TIM that had substantially lower thermal resistance than the plain polymer due to highly efficient thermal conduction pathways within the poorly conductive polymer matrix [93,94]. Their high thermal conductivity and flexibility allow the coating to conform to rough surfaces by filling in 5 microscopic gaps between components and sinks which can improve device performance and longevity [87].

Furthermore, MXenes have been proposed as components of thermal protection or barrier systems. Their high-temperature stability in inert atmospheres suggests they could serve as protective layers in extreme thermal environments [95]. For example, a coating of a MAX or MXene on a metal could act as an oxidation-resistant barrier up to 800–1000°C [81]. Also, their high emissivity (due to their electronic structure) means they radiate heat effectively at high temperatures, which can be useful for passive cooling (MXene coatings could enhance radiative heat dissipation of a hot surface) [81].

Taken together, MXenes can be regarded as in-plane thermal conductors and through-plane thermal insulators. Among materials, this combination is rare and particularly valuable for advanced thermal management applications. Their ability to withstand elevated temperatures in inert environments, coupled with tunable thermal transport through compositional and surface-termination control, enables use across a broad temperature and application space. These characteristics enable both heat-spreading and thermal insulation functions depending on material orientation, density, and processing route. For example, MXene aerogels exhibit ultra-low thermal conductivities as low as ≈0.017~0.025 W·m^−1^·K^−1^ [96,97], comparable to air and commercial insulators, while dense, well-aligned MXene films and composites can achieve in-plane thermal conductivities achieving up to 42 W·m^−1^·K^−1^ or higher significantly exceeding typical polymer matrices. This wide tunability through structural orientation and density control is particularly attractive for mechanically integrated thermal solutions.

### 5.2. Barrier Properties (Gas and Moisture)

**Figure 8:**
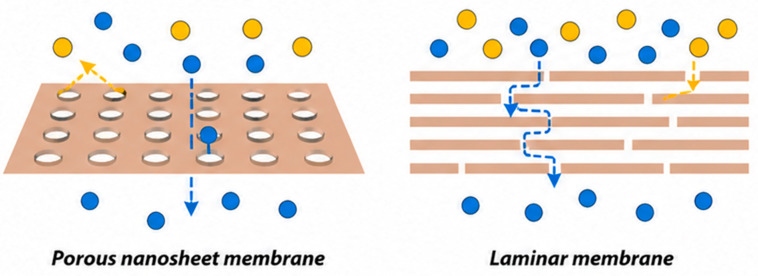
Two types of two-dimensional-material membranes: porous nanosheet membranes and laminar membranes. Reproduced with permission from [100].

MXenes can form dense, highly aligned laminar films that exhibit low gas permeability when processed appropriately. In such structures, gas molecules must navigate a complex diffusion path through stacked two-dimensional flakes, significantly reducing the effective transport rate as shown in Figure 8 [98]. Importantly, this barrier behavior reflects macroscopic diffusion resistance through the film rather than the chemical inertness of the MXene itself. Although MXenes, particularly Ti_3_C_2_T_x_, are chemically susceptible to oxidation, especially in humid or oxygen-rich environments [99], dense MXene films can nevertheless display low oxygen transmission rates due to their tightly packed layered architecture [98].

A study on MXene-reinforced PBAT/Ti_3_C_2_T_x_ composite films shows that adding Ti_3_C_2_T_x_ nanosheets decreases the oxygen transmission rate (OTR) of the films compared with the neat polymer, consistent with the idea that aligned MXene sheets increase the effective diffusion path and improve gas barrier properties. Specifically, incorporation of 1 wt % Ti_3_C_2_T_x_ into PBAT reduced OTR from ≈1030 cc·m^−2^·day^−1^ for pristine PBAT to ≈782 cc·m^−2^·day^−1^ in the composite film, and biaxial stretching further reduced OTR to ≈732 cc·m^−2^·day^−1^ due to enhanced orientation of filler and polymer [101]. While not yet matching the ultra-low oxygen transmission rates of aluminum foil or inorganic coatings [102], these values represent substantial improvements over conventional polymers and highlight the potential of MXenes as thin, multifunctional oxygen-barrier layers.

In contrast to their resistance to gas diffusion, pristine MXene films generally exhibit poor water vapor barrier performance. This limitation arises from the hydrophilic nature of MXene surface terminations (e.g., -OH, -O, -F), which readily absorb moisture and promote interlayer swelling, reducing the effectiveness as a moisture barrier [103]. Consequently, MXenes are rarely employed as standalone materials in applications requiring simultaneous resistance to both oxygen and water vapor.

Several strategies have been explored to address this limitation, including incorporation of MXenes into hydrophobic polymer matrices, application of moisture-resistant overcoatings, and chemical modification of MXene surfaces to reduce hydrophilicity [104,105,106]. In some systems, moderate humidity has been observed to temporarily enhance oxygen barrier performance by swelling the layered structure and closing microscale defects reducing gas permeation pathways [103]. However, excessive moisture ultimately degrades barrier performance by introducing continuous vapor transport channels through the film.

Beyond packaging applications, MXene barrier behavior is also relevant to corrosion protection. Dense MXene-based coatings can limit the ingress of oxygen and water to underlying metal substrates, delaying corrosion initiation under controlled exposure conditions [107]. While such coatings do not provide long-term chemical passivation, reductions in corrosion rate (on the order of tens of percent) have been reported in salt spray and electrochemical testing environments, highlighting the potential of MXenes as diffusion-barrier components in protective coating systems [108,109].

### 5.3. Tribological (Friction and Wear) Performance

MXenes, owing to their two-dimensional layered morphology, are frequently compared to established solid lubricants such as graphite and MoS_2_ [110]. Their structure allows adjacent layers to shear relative to one another under load, resulting in low interfacial shear strength and reduced friction [110]. Unlike purely van der Waals-bonded materials, MXene layers also possess surface terminations (e.g., -OH, -O, -F), which influence interlayer interactions and tribological behavior.

Experimental studies have shown that Ti_3_C_2_T_x_ MXene can significantly reduce friction in tribological contacts, but the magnitude of this reduction depends strongly on materials system, environment, and contact mechanics. As lubricant additives, Ti_3_C_2_T_x_ at low weight fractions (~0.3 wt%) in tailored ionic liquid or composite lubricant formulations has achieved friction coefficients around 0.06–0.07 in steel-steel contacts, representing a substantial reduction compared to base oils [111]. Hybrid solid coatings combining Ti_3_C_2_T_x_ with other 2D materials have also yielded COF values near 0.065 under ambient ball-on-disc tests [112]. Water-based lubricant additives typically produce more moderate reductions (e.g., ~0.20–0.26 from ~0.29–0.33 for ~5 wt% Ti_3_C_2_) [113]. Nanoscale AFM measurements indicate friction coefficients below 0.01 at atomic scales, but such values should not be taken as representative of macroscale tribological performance [114].

MXenes have also been investigated as lubricant additives in both oil-based and water-based systems, typically at low concentrations (between 0.1 wt% to 5 wt%) [110]. When dispersed in base fluids, MXene flakes can migrate to contact interfaces and form protective tribofilms that reduce friction and wear [115]. Reported results include significant reductions in friction coefficients and wear volumes relative to the neat lubricant, particularly under low-to-moderate loads and sliding speeds and the effectiveness of MXenes in such roles depends strongly on dispersion quality, concentration, and operating regime [110].

A notable advantage of MXenes as lubricant additives is their hydrophilic surface chemistry, which enables stable dispersion in polar lubricants such as water-glycol mixtures [116]. This contrasts with graphite, which disperses poorly in aqueous systems [117]. MXenes can also function effectively under humid conditions, whereas the lubricity of graphite is highly sensitive to environmental moisture content [117].

MXene-based surface coatings have additionally demonstrated wear-reduction capability. Thin Ti_3_C_2_T_x_ coatings deposited on metallic substrates have been shown to reduce wear scar depth and area during sliding contact, primarily through the formation of a continuous, shearable tribofilm [118]. While MXenes are mechanically stiffer than graphite, their tribological benefit arises from interfacial film formation and shear accommodation rather than bulk load bearing [118].

Environmental stability remains an important consideration. During sliding in air, MXenes may undergo partial oxidation, leading to the formation of oxide species such as TiO_2_ within the tribofilm [115]. Depending on thickness and distribution, such oxides can either contribute to wear resistance or degrade lubricity. Consequently, MXenes generally exhibit more stable tribological performance in lubricated or controlled environments than in dry, oxidative conditions [110].

Relative to traditional solid lubricants, MXenes offer a distinct combination of properties. Graphite performs well in humid air but poorly in dry or vacuum environments, while MoS_2_ excels in vacuum yet degrades in humid air due to oxidation [110]. MXenes, although also susceptible to oxidation, provide tunable surface chemistry and can be continuously supplied to contact interfaces as lubricant additives, potentially mitigating some environmental limitations. Their electrical conductivity further enables charge dissipation in tribological contacts, which may be beneficial in electrically sensitive mechanical systems.

Overall, MXenes represent a promising class of tribological materials whose friction and wear performance can be tailored through surface chemistry, additive concentration, and operating environment. While many reported results remain system-specific, the ability of MXenes to achieve low friction and effective wear reduction through tribofilm formation positions them as attractive candidates for next-generation lubricants and coatings in mechanically demanding applications.

### 5.4. Electrical and Electrochemical Behavior

Beyond their mechanical and tribological relevance, MXenes exhibit unusual electrical and electrochemical behavior that separates them from most structural and coating materials. Unlike many 2D materials, MXenes are intrinsically metallic or semi-metallic, with electrical conductivities that can rival or exceed those of traditional conductive coatings. This property is especially notable because it coexists with high surface area, layered morphology, and tunable surface chemistry [113].

Most studied MXenes, such as Ti_3_C_2_T_x_, show room-temperature electrical conductivities on the order of 10^3^–10^4^ S/cm in well-processed films, depending strongly on flake size, alignment, defect density, and surface terminations [105]. These values place MXenes closer to metals and conductive carbons than to semiconducting 2D materials like MoS_2_. For mechanical engineers, this means MXenes can function simultaneously as load-bearing or protective layers and as electrically conductive elements, which is uncommon in conventional coatings.

The electrical behavior of MXenes is highly sensitive to their surface terminations (-O, -OH, -F), which are introduced during synthesis. In practical terms, electrical performance is not a fixed material constant but a tunable property, adjustable through processing and post-treatment. Mechanical deformation, such as bending or sliding, can also alter interlayer contact and thus conductivity, introducing the possibility of strain- or wear-dependent electrical response [113].

Electrochemically, MXenes behave as pseudocapacitive materials rather than purely electrostatic ones. Their surfaces readily participate in fast, reversible redox reactions with ions in contact media, enabled by their metallic conductivity and accessible surface terminations. While this behavior is most often discussed in the context of energy storage, the underlying physics (fast surface charge transfer, ion adsorption, and redox activity) have broader implications [119]. From a mechanical perspective, these electrochemical interactions can influence corrosion behavior, interfacial charge buildup, and tribo-electrical effects during sliding contact [119].

An important consideration is environmental sensitivity. MXenes can oxidize over time, especially in humid or aqueous environments, leading to changes in both electrical conductivity and electrochemical response [120]. Oxidation typically converts surface titanium into TiO_2_, which is electrically insulating [120]. However, partial oxidation does not always eliminate functionality and may even stabilize certain interfaces, depending on the application environment. This highlights a recurring theme in MXene behavior: performance is strongly coupled with operating conditions and material history.

Overall, MXenes occupy a unique space among engineering materials by combining metallic-level electrical conductivity with layered, mechanically compliant structures and chemically active surfaces. For mechanical engineers, this multifunctionality opens opportunities where mechanical performance must coexist with electrical conduction or electrochemical interaction. Understanding how processing, environment, and mechanical loading influence these properties is essential before MXenes can be reliably integrated into mechanically demanding systems.

## 6. Applications in Mechanical Engineering

MXenes’ combination of mechanical strength, conductivity, and surface reactivity opens a wide array of applications in mechanical engineering and related fields. Here we highlight several key areas where MXenes are being applied or have strong potential: (6.1) nanocomposites for mechanical reinforcement, (6.2) integrated energy storage components, (6.3) sensors and actuators, (6.4) thermal management devices, and (6.5) protective coatings and membranes. Table 5 provides an overview of some representative MXene applications, their demonstrated benefits, and current technology readiness levels (TRL).

### 6.1. Mechanical Reinforcement and Structural Composites

One of the most immediate and promising applications of MXenes in mechanical engineering is their use as reinforcing additives in structural composites. Owing to their extremely high aspect ratio, high intrinsic stiffness, and chemically active surface terminations, MXenes function as effective two-dimensional nanofillers capable of significantly enhancing the mechanical performance of polymers and fiber-reinforced composites at relatively low loadings [92]. One of the most immediate and promising applications of MXenes in mechanical engineering is their use as reinforcing additives in structural composites. Owing to their extremely high aspect ratio, high intrinsic stiffness, and chemically active surface terminations, MXenes function as effective two-dimensional nanofillers capable of significantly enhancing the mechanical performance of polymers and fiber-reinforced composites at relatively low loadings [92].

In polymer matrices, MXenes can be viewed as “2D fibers” dispersed within the matrix. Even at loadings of only a few weight percent, MXene additions have been shown to substantially improve stiffness, strength, and toughness. These enhancements arise from several complementary mechanisms:

- Efficient load transfer:

MXene sheets are mechanically stiff and can bear load provided stress is effectively transferred from the surrounding matrix. The abundant surface terminations (-O, -OH, -F) on MXenes promote strong interfacial interactions with polar polymers through hydrogen bonding or, in some systems, covalent bonding enabled by coupling agents or cross-linking. As a result, applied stress is efficiently transferred from the polymer matrix to the MXene reinforcement, leading to increased modulus and strength [129].

- Crack deflection and bridging:

Thin MXene flakes act as obstacles to crack propagation. Cracks may be deflected along MXene-matrix interfaces or bridged by MXene sheets spanning the crack faces, delaying crack growth and increasing fracture resistance. This mechanism contributes to improved toughness and reduced catastrophic failure [130].

- Energy dissipation:

The introduction of MXenes creates additional internal interfaces within the composite. Under mechanical loading (particularly cyclic or impact loading), interfacial sliding between MXene sheets and the matrix, or between adjacent MXene layers, can dissipate energy. This behavior is analogous to the layered energy-dissipation mechanisms observed in natural nacre and contributes to enhanced damage tolerance [131].

As a result of these mechanisms, substantial mechanical property improvements have been reported. For example, Ti_3_C_2_T_x_/epoxy composites have demonstrated tensile strength increases on the order of ~50% relative to neat epoxy, accompanied by increases in elastic modulus and toughness (Table 5) [132,133]. Importantly, these gains are achieved without the severe embrittlement often observed with traditional rigid fillers.

MXenes have also proven particularly effective as interfacial modifiers in fiber-reinforced composites. In carbon fiber/epoxy systems, coating carbon fibers with a thin layer of Ti_3_C_2_T_x_ MXene has resulted in tensile strength increases of approximately 100% and flexural strength increases of roughly 67% [133]. In such systems, the MXene improves interfacial bonding between the smooth carbon fiber surface and the epoxy matrix while also contributing localized reinforcement in resin-rich regions [133].

An additional advantage of MXene-reinforced composites is their inherent multifunctionality. At sufficient loadings, MXenes can form electrically conductive networks within otherwise insulating polymer matrices [134]. This enables the development of structural composites that also exhibit electrical conductivity for functions such as electromagnetic interference (EMI) shielding, Joule heating, or self-sensing [135]. In particular, MXene-based composites often display piezoresistive behavior, where electrical resistance changes with mechanical deformation, allowing the material to act as an embedded strain sensor [136].

Beyond polymers, MXene reinforcement of metals and ceramics is an active but early stage area of research. In metal matrix composites, MXenes have been incorporated via powder mixing followed by low-temperature consolidation to limit MXene degradation, with exploratory work reported in systems such as MXene/Cu composites [137]. For ceramics, MXenes are being investigated in cold sintering and laminated architectures to enhance fracture toughness through crack deflection mechanisms like those used with other platelet reinforcements [138].

A notable advantage of MXenes compared to many conventional fillers is their ability to improve stiffness and toughness simultaneously. This capability addresses a longstanding challenge in composite design, where increases in stiffness often come at the expense of fracture resistance [138]. MXene hybrid composites (by combining MXenes with traditional fibers or other nanomaterials such as carbon nanotubes) further expand this design space by leveraging their independent yet complementary reinforcement mechanisms [138].

Overall, MXenes represent a highly promising class of reinforcing agents for next-generation structural composites, offering a rare combination of mechanical reinforcement, damage tolerance, and functional integration that is well aligned with emerging mechanical engineering design requirements.

### 6.2. Energy Storage and Electromechanical Integration

MXenes are extensively studied for electrochemical energy storage, particularly in supercapacitors and batteries. From a mechanical engineering perspective, a particularly compelling direction is structural energy storage, in which load-bearing components simultaneously store electrical energy. MXenes are well suited to this concept because they combine high electrical conductivity, high charge storage capability, and mechanically robust, processable film architectures [139].

One of the most mature demonstrations involves flexible MXene-based supercapacitors. Free-standing Ti_3_C_2_T_x_ MXene films can function simultaneously as the active electrode material and current collector, eliminating the need for metallic foils or polymer binders [140]. These films, typically on the order of 10–50 μm thick, exhibit good flexibility and can tolerate moderate tensile strains and repeated bending without loss of electrical performance or capacitance [140]. As a result, MXene supercapacitors can be integrated into flexible or conformal components, such as structural skins or housings, where they provide distributed energy storage.

MXene electrodes exhibit exceptional electrochemical performance. Gravimetric capacitances exceeding 350 F·g^−1^ have been reported, with approximately 90% capacitance retention after 10,000 charge–discharge cycles (Table 5) [122,123]. Their high electrical conductivity and thin ion diffusion pathways enable rapid charge and discharge, making MXene-based supercapacitors particularly attractive for high-power pulse applications, such as regenerative energy capture, power smoothing, or short-duration actuator operation.

Beyond standalone devices, structural power composites have been explored in which MXene layers are integrated into fiber-reinforced polymer architectures. In these concepts, conventional fibers (e.g., carbon fibers) carry the primary mechanical loads, while MXene layers serve as electrochemically active elements embedded within the composite. Solid or gel polymer electrolytes can be incorporated into the matrix, allowing the structure to function as a supercapacitor or battery-like device [141]. Although these systems remain at the laboratory or early prototype stage, they illustrate the potential to reduce system-level mass by eliminating discrete energy storage components.

MXenes have also been investigated as electrode materials for lithium-ion and other battery chemistries, where their high conductivity and layered structure enable high-rate capability [142]. While structural batteries based on MXenes are still largely conceptual, ongoing research aims to develop multifunctional components (such as casings or panels) that contribute both mechanically and electrochemically.

An additional advantage of MXenes is their processability. Their hydrophilic surface chemistry allows fabrication using aqueous electrolytes and water-based processing routes, offering environmental and manufacturing advantages over many carbon-based electrode materials [1]. Furthermore, MXene films are self-supporting and self-adhering, simplifying electrode fabrication and integration [143].

Overall, MXenes provide a unique pathway for integrating electrical energy storage directly into mechanical components. While challenges remain in balancing mechanical performance, electrochemical stability, and long-term durability, MXene-based structural energy storage devices have already reached prototype-level maturity and represent a promising direction for multifunctional mechanical systems.

### 6.3. MXene-Based Sensors and Actuators

MXenes are increasingly explored for sensing and actuation applications due to their high electrical conductivity, large accessible surface area, mechanical flexibility, and chemically active surfaces. These characteristics enable strong coupling between mechanical deformation, environmental stimuli, and electrical response, which is particularly valuable for mechanically integrated sensing and soft actuation systems [144].

#### 6.3.1. Mechanical Sensors (Strain and Pressure)

MXene films and MXene-based composites commonly function as piezoresistive sensors, in which applied strain or pressure alters the conductive pathways within the material, leading to measurable changes in electrical resistance [144]. In thin-film or elastomer-supported configurations, deformation modifies interflake contact resistance, tunneling distances, and network connectivity [145].

MXene-based strain sensors have demonstrated high sensitivity, with the ability to detect strains below 0.2% and maintain stable operation over thousands of loading cycles (Table 5) [124]. Reported gauge factors vary widely depending on material architecture, ranging from tens in dense films to several thousand or higher in porous or advanced microstructured composites [146,147]. MXene coatings on elastomeric substrates such as PDMS can function as flexible strain sensors capable of large deformation, while maintaining repeatable electrical response [147,148].

**Figure 9:**
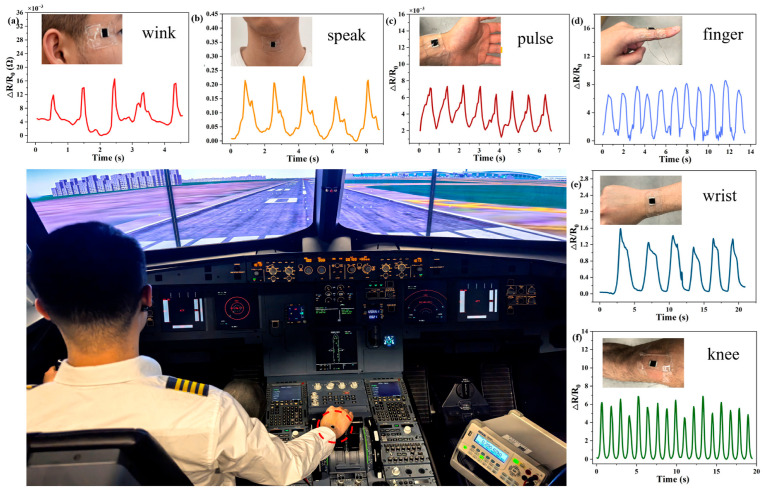
Relative resistance change (ΔR/R) signals of the flexible strain sensor under different human motions: (**a**) eye blinking; (**b**) speaking; (**c**) pulse monitoring; (**d**) finger bending; (**e**) wrist movement; and (**f**) knee movement. Reproduced from [147], Open access.

Pressure sensing can be achieved using porous MXene foams or aerogels, where compressive loading increases interflake contact and reduces overall resistance [149]. These structures exhibit high pressure sensitivity at low loads and good cyclic durability, allowing them to sense pressure from gentle breezes and human motion (e.g., small-scale pulse beating and large-scale walking and running) [150,151]. This makes them attractive for mechanical engineering purposes like tactile sensing and distributed pressure monitoring. A key advantage of MXenes in mechanical sensors is their ease of processing: continuous conductive networks can be formed via simple dip-coating, spraying, or printing of MXene inks onto fabrics, polymers, or foams [36,152]. The practical sensitivity of MXene-based strain sensors in detecting subtle and large-scale mechanical deformation is illustrated in Figure 9. Similar flexible, skin-mounted sensing platforms can continuously monitor physiological and motion-induced strain by translating deformation into real-time resistance changes, demonstrating applicability in dynamic environments such as human motion tracking and pilot health monitoring [147].

#### 6.3.2. Environmental Sensors (Humidity, Gas, Temperature)

MXenes are also responsive to environmental stimuli due to their surface terminations and interlayer structure. Humidity sensing is particularly well established: water adsorption leads to interlayer swelling and changes in charge transport, resulting in measurable resistance shifts [103]. Ti_3_C_2_T_x_-based humidity sensors have demonstrated high sensitivity and response times as low as 3.86 s at 91.5% relative humidity, though long-term stability depends on oxidation control [153].

In gas-sensing applications, MXenes demonstrate exceptional sensitivity to ammonia (NH_3_) across the 0.5–100 ppm range, while maintaining the capacity to detect volatile organic compounds (VOCs) at trace concentrations as low as 50–100 ppb at room temperature [154,155]. Compared to conventional carbon-based nanomaterials, MXenes yield higher signal amplitudes; their abundant surface functional groups, which facilitate stronger molecule-surface interactions and more efficient charge transfer [155]. However, the long-term operational lifespan of these sensors is often limited by environmental degradation and irreversible gas adsorption, both of which alter carrier concentration and baseline conductivity [156].

MXene-based temperature sensors utilize the material’s intrinsic temperature-dependent electrical resistance to provide high-resolution thermal monitoring. While pure MXene films typically exhibit a metallic positive temperature coefficient (PTC), their sensitivity can be significantly amplified through architectural engineering [157]. In 2025, research in Nature identified that water molecules confined between MXene layers can trigger a reversible metal-to-semiconductor transition, inducing large resistance changes over modest temperature ranges [157]. Furthermore, when integrated into flexible or conformal composite (e.g., MXene-enhanced hydrogels or elastomers), the sensitivity is further boosted by the thermal expansion of the host matrix, which modifies interlayer contact resistance [158]. Recent studies have demonstrated that these architectures can achieve sensitivities as high as 3244% °C^−1^, enabling their application in ”electronic skins” that maintain repeatable electrical responses while conforming to irregular surfaces [158].

#### 6.3.3. Multifunctional Sensing

Because MXenes respond electrically to multiple stimuli, a single MXene-based sensor can often detect strain, pressure, humidity, or temperature simultaneously. By analyzing distinct response signatures (e.g., resistance magnitude, rate of change, or frequency dependence), multifunctional sensing platforms can be utilized for electronic skin and structural monitoring applications [159,160].

#### 6.3.4. Actuation and Electromechanical Response

Although MXene-based actuators are currently less mature than their sensing counterparts, several sophisticated actuation strategies have emerged. In electroactive hydrogels, MXene sheets function as high-conductivity frameworks that facilitate electrically driven ionic migration and asymmetric swelling, leading to precise mechanical bending or deformation [161]. Recent breakthroughs in light-driven systems include stretchable MXene-based hydrogel actuators capable of bending approximately 360° under 808 nm NIR irradiation, while maintaining a robust maximum peel force of 280 N m^−1^, demonstrating significant potential for light-controlled soft robotics [162].

Beyond ionic systems, MXenes are also effective Joule heating elements due to their exceptional electrical conductivity and thin-film geometry [163]. When integrated into bilayer structures with thermoresponsive polymers, applied current triggers rapid, uniform heating that leverages the mismatch in coefficients of thermal expansion (CTE) between the layers to drive thermal actuation [164]. Furthermore, the mechanical resilience of MXene networks allows them to serve as compliant electrodes in soft robotics, where high stretchability and stable electrical contact are required to maintain performance under large-scale mechanical strains [165].

#### 6.3.5. Outlook and Maturity

Overall, MXene-based sensors have reached laboratory to early prototype readiness, with demonstrated sensitivity, mechanical durability, and processing scalability. Actuation applications remain more exploratory but highlight the potential of MXenes to serve as multifunctional components that combine sensing, electrical conduction, and mechanical compliance within integrated mechanical systems.

### 6.4. Thermal Management and Heat Dissipation

The thermal transport characteristics of MXenes, discussed in Section 5.1, translate into several promising applications in thermal management systems. Their combination of high in-plane thermal conductivity, low through-plane conductivity, mechanical flexibility, and processability enables multifunctional solutions for heat spreading, thermal interfacing, insulation, and electromagnetic compatibility.

#### 6.4.1. Heat Spreaders

Thin MXene-based films have been explored as lateral heat spreaders for electronic and electromechanical components. Due to strong in-plane bonding within MXene sheets, heat can be efficiently redistributed across a surface, reducing localized thermal gradients and hotspot formation. Hybrid films combining MXenes with other high-conductivity fillers, such as graphene, have demonstrated in-plane thermal conductivities in the range of approximately 26–37 W·m^−1^·K^−1^ (Table 5) [125]. These values exceed those of typical polymer films and approach those required for effective heat-spreading layers in compact electronic assemblies. Experimental demonstrations at the device or module level have shown measurable reductions in peak surface temperature through improved lateral heat distribution [125].

#### 6.4.2. Thermal Interface Materials (TIMs)

MXenes have also been incorporated into thermal interface materials, where they serve as conductive fillers within polymer matrices. Their two-dimensional geometry enables the formation of efficient conductive networks at low filler concentration, while their flexibility allows them to conform to microscale surface roughness between contacting solids [93,94]. This combination reduces interfacial thermal resistance and improves overall heat transfer. MXene-based TIMs have demonstrated significantly improved thermal conductivity and lower contact resistance compared to unfilled polymers, highlighting their potential for use as soft pads, adhesives, or coatings in thermal interfaces [93].

#### 6.4.3. MXene-Metal Hybrid Systems for Extreme Environment and Thermal Applications

MXenes have also been investigated in hybrid systems incorporating metallic particles (e.g., Ag, Cu, Al), where synergistic effects enable enhanced thermal transport and environmental robustness under extreme conditions. In these systems, metal particles act as high-conductivity bridges between MXene flakes, reducing interfacial thermal resistance and improving through-plane heat transfer, which is otherwise limited in layered MXene assemblies. This hybridization can increase overall thermal conductivity while maintaining the processability and interfacial compatibility of MXenes [166,167].

Recent studies demonstrate that MXene–metal composites exhibit improved thermal stability at elevated temperatures compared to pristine MXenes, as metallic phases can retard oxidation by acting as diffusion barriers or sacrificial oxidation sites. Additionally, these composites maintain thermal conductivity under cyclic heating and high heat flux conditions, making them promising for applications in harsh environments such as aerospace components, high-power electronics, and thermal protection systems [168].

In extreme environments, including high-temperature oxidative atmospheres and rapid thermal cycling, MXene–metal systems have shown enhanced durability relative to polymer-based composites. The incorporation of metallic fillers not only improves heat dissipation but also contributes to structural integrity under thermomechanical stress. However, challenges remain in controlling interfacial bonding, preventing galvanic effects, and optimizing filler dispersion to avoid agglomeration and thermal short-circuiting. These findings highlight MXene-metal hybridization as a viable pathway toward extending MXene-based thermal management technologies into demanding operational environments where both high thermal conductivity and environmental resilience are required [166,168].

#### 6.4.4. Thermal Shields and Insulation

**Figure 10:**
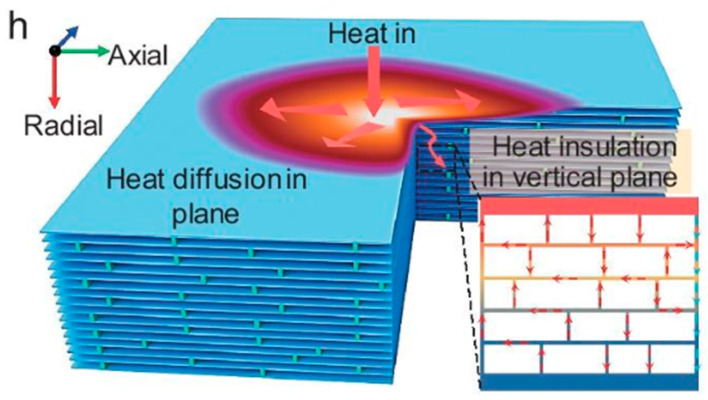
Schematic representation of heat transfer within a porous MXene aerogel, reducing thermal transport through scattering, interfacial resistance, and restricted conduction pathways. Reproduced from [96], Open access.

At the opposite end of the thermal spectrum, MXene aerogels and foams have been investigated as thermal insulating materials. Their porous, low-density structures disrupt heat conduction, yielding effective thermal conductivities comparable to conventional insulation materials [88,96]. In addition, MXene-containing coatings can serve as thermal protection layers, where controlled oxidation or structural degradation under high heat flux may contribute to sacrificial protection of the underlying substrate. Such concepts are particularly relevant for fire-resistant panels and thermally protective composites, though long-term oxidation stability remains a key challenge. The insulating behavior of MXene-based aerogels is closely tied to their porous structure, which disrupts heat flow across multiple length scales. As illustrated in Figure 10, this architecture limits heat transfer by impeding conduction pathways, enabling effective thermal insulation despite the intrinsically conductive nature of individual MXene sheets [93].

#### 6.4.5. Coupled EMI Shielding and Thermal Management

MXenes are well known for their electromagnetic interference (EMI) shielding capability due to their high electrical conductivity. Importantly, this functionality can be combined with thermal management. MXene-based coatings and composites have demonstrated EMI shielding effectiveness on the order of 24–92 dB, while simultaneously providing thermal conduction and, in some cases, barrier protection [126,127]. This multifunctionality is particularly attractive for electronic packaging, where both electromagnetic compatibility and heat dissipation are required within a limited space.

#### 6.4.6. Phase Change Material (PCM) Enhancement

MXenes have been incorporated into phase change materials to enhance their thermal conductivity. By facilitating faster heat transfer into and out of the PCM, MXene additives improve the charging and discharging rates of thermal energy storage systems [169]. This is demonstrated by a study that has shown an increase in thermal conductivity of up to 708% compared to traditional composites [169]. This approach is relevant for passive cooling applications, where rapid thermal response is critical to maintaining temperature control.

#### 6.4.7. Outlook and Design Considerations

The strong anisotropy of MXene thermal transport enables design strategies that combine lateral heat spreading with through-thickness insulation [87]. While MXene flakes naturally align parallel to surfaces during processing, controlling their orientation remains an active area of research. Hybrid systems combining MXenes with one-dimensional fillers, such as carbon nanotubes, have shown synergistic improvements by bridging interlayer thermal gaps and enhancing overall heat transport [170].

Overall, MXene-based thermal management solutions have reached laboratory to pilot-scale demonstration. Continued progress will depend on improving oxidation resistance, long-term thermal stability, and scalable integration with existing thermal management architectures.

### 6.5. Functional Coatings and Membranes

MXenes can be processed into thin coatings or layered membranes that impart multiple surface-level functions without significantly altering bulk material properties. This capability makes them attractive as multifunctional surface treatments in mechanical engineering systems.

EMI shielding coatings are among the most mature MXene-based applications. Spray- or paint-applied Ti_3_C_2_T_x_ coatings on polymers, foams, or textiles routinely achieve electromagnetic shielding effectiveness on the order of ~24–92 dB in the GHz frequency range at micrometer-scale thicknesses [126]. When normalized by weight and flexibility, this performance is comparable to metallic foils, while offering advantages in corrosion resistance, conformability, and lightweight integration [109]. Such coatings are suitable for electronic enclosures, cables, and structural components requiring EMI mitigation, and are being explored for aerospace and transportation systems.

Corrosion-inhibiting coatings based on MXene films provide protection primarily through barrier mechanisms, limiting the ingress of oxygen and moisture to underlying metals [126]. Studies on steel and aluminum substrates show that MXene coatings can measurably extend corrosion lifetimes in salt-spray and immersion environments [109]. In addition, partial oxidation of Ti-based MXenes to form surface TiO_2_ may further reduce local oxygen availability, contributing to corrosion suppression [171]. While galvanic interactions depend on substrate and environment, MXenes have demonstrated compatibility with common structural metals under controlled conditions [172].

Wear-resistant and low-friction coatings represent another promising direction. When incorporated into polymer matrices or applied as thin surface layers, MXenes reduce friction coefficients and wear rates by acting as solid lubricants at the sliding interface [111]. For example, MXene-polymer composite coatings on aluminum alloys combine load-bearing matrices with lubricious MXene layers, improving tribological performance without sacrificing structural integrity [117].

MXene membranes leverage the layered morphology and tunable interlayer spacing of MXenes to enable selective transport of molecules or ions. Research has demonstrated high water permeability and effective ion sieving in Ti_3_C_2_T_x_ membranes, with performance exceeding that of graphene oxide membranes in comparable laboratory tests [173]. While MXene membranes have not yet matched commercial reverse osmosis standards for desalination, they show strong potential for nanofiltration, solvent separation, pervaporation, and controlled ion transport, particularly where high flux and surface functionality are required [128].

An emerging concept is the development of “smart” membranes, enabled by the intrinsic electrical conductivity of MXenes. Because transport and fouling processes affect electrical resistance and capacitance, MXene membranes can function simultaneously as filters and self-monitoring sensors [174]. In addition, applied electrical bias has been explored as a means of reducing fouling or inhibiting biofilm formation through electrochemical effects, although these approaches remain at an early research stage [174].

MXenes also show promise in solid-lubricant coatings for specialized environments such as vacuum or inert atmospheres. Under sliding contact, MXene layers can shear and reorient, providing low-friction behavior analogous to other layered lubricants [175]. However, environmental stability remains a key limitation, as oxidation in air can degrade lubricity, restricting applicability to controlled environments [175].

From a manufacturing perspective, MXene-based coatings are highly scalable. Stable aqueous inks can be spray-coated, printed, or deposited via roll-to-roll processes, enabling large-area coverage on complex geometries [176]. Similar scalable approaches apply to membrane fabrication using porous supports. Beyond mechanical and transport functions, MXenes have also been incorporated into polymer systems as flame-retardant additives, where they promote char formation and thermal shielding, improving fire resistance in fabrics and foams [177].

Overall, MXenes enable multifunctional coatings and membranes that combine electrical conductivity, EMI shielding, corrosion resistance, lubrication, and selective transport within a single material layer. This convergence of functions is particularly attractive for mechanical engineering systems, where reducing component count, weight, and assembly complexity is often as critical as improving individual material properties.

## 7. Challenges and Future Directions

While MXenes show enormous promise, several challenges must be addressed to fully realize their potential across industries. Key areas for future research include: improving stability (especially against oxidation), scaling up production economically and safely, integration into existing manufacturing and material systems, and establishing standards and reproducibility for MXene materials [117]. We discuss these challenges and emerging strategies to overcome them.

### 7.1. Stability, Moisture Sensitivity, and Durability Mechanisms and Mitigation

**Figure 11:**
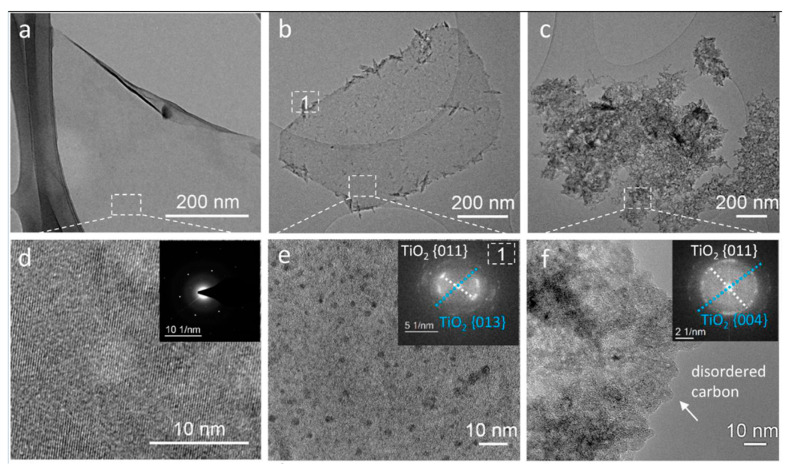
Transmission electron microscopy images of the structural evolution of Ti_3_C_2_T_x_ MXene flakes during ambient aging: (**a**) pristine flakes with clean surfaces, (**b**) partially oxidized flakes after 7 days exhibiting edge growths and nanoparticle formation, and (**c**) fully degraded material after 30 days. Corresponding high-resolution images (**d**–**f**) reveal crystalline features in the pristine state and progressive transformation into oxide phases upon aging. Reproduced with permission from [99].

A primary limitation of MXenes, particularly Ti-based compositions such as Ti_3_C_2_T_x_, is their susceptibility to oxidation and environmental degradation. Exposure to air or aqueous environments leads to gradual formation of surface oxides and hydroxides, resulting in diminished electrical conductivity and altered surface chemistry [99]. This progressive structural degradation is directly observable at the nanoscale, as shown in Figure 11, where initially well-defined MXene flakes evolve into oxide-decorated and ultimately decomposed structures under ambient aging conditions [99]. Quantitative studies indicate that Ti_3_C_2_T_x_ aqueous suspensions can exhibit measurable oxidation within days and substantial degradation within 1–2 weeks under ambient conditions, while freestanding films stored in air may retain functionality for several weeks to months depending on flake size, defect density, and humidity [82]. This instability reduces shelf life, processing windows, and long-term device reliability, making improved environmental stability a critical requirement for practical deployment.

Degradation is governed by coupled oxygen diffusion and defect-mediated reaction pathways, where edge sites, vacancies, and -OH terminations act as active centers for TiO_2_ nucleation. Oxidation kinetics are accelerated in smaller flakes and defect-rich structures due to increased surface area and reactivity, while multilayer films exhibit comparatively slower, diffusion-limited degradation behavior. These degradation mechanisms are strongly dependent on composition, surface terminations, and morphology. Ti-based MXenes generally exhibit lower oxidation resistance compared to Mo- or V-based systems due to differences in metal–oxygen affinity and oxide stability. Surface terminations further modulate reactivity, with –OH and -O groups promoting oxidation pathways, while –F terminations provide partial passivation. Morphologically, single- and few-layer flakes degrade more rapidly than multilayer stacks due to increased exposure of reactive edge and basal sites. These mechanisms directly impact mechanical performance, leading to embrittlement, reduced interlayer cohesion, and progressive loss of load transfer capability in structural applications [82,178].

Several stabilization strategies have been explored. Encapsulation approaches physically isolate MXenes from oxygen and moisture using protective barrier layers. Polymer coatings such as parylene or epoxy, as well as lamination between impermeable two-dimensional materials (e.g., graphene or hexagonal boron nitride), have been shown to significantly slow oxidation [179]. For example, ultrathin atomic-layer-deposited (ALD) Al_2_O_3_ coatings (≈2–10 nm) act as effective diffusion barriers, enabling conductivity retention over extended periods under ambient exposure and improving resistance to humidity-driven degradation [179].

Chemical stabilization during storage is another effective route. Antioxidant additives such as sodium ascorbate can scavenge dissolved oxygen in aqueous MXene dispersions, markedly slowing degradation [180]. Such treatments have been shown to extend suspension lifetimes from days to several months when combined with oxygen-free environments (e.g., argon-filled containers) and low-temperature storage [180].

More fundamentally, intrinsic modification of MXene chemistry is being investigated to improve oxidation resistance. Elemental doping and compositional tuning, such as boron incorporation into Ti_3_C_2_T_x_, have demonstrated enhanced resistance to environmental degradation through suppressing oxygen diffusion and improve surface polarity [181]. Other approaches, including silicon incorporation or engineered passivation layers formed during synthesis, remain exploratory but highlight promising directions for stabilizing MXenes at the atomic level [182].

Moisture sensitivity presents a related challenge. While hydrophilicity enables excellent dispersion and processability, it can be detrimental in applications requiring dimensional, electrical, or baseline stability under varying humidity [106]. Surface functionalization strategies, such as salinization to introduce hydrophobic groups, have been employed to reduce moisture uptake [183]. However, fully hydrophobic MXenes are difficult to achieve due to their intrinsically polar nature, making composite architectures or encapsulated micro-environments a more practical solution [179].

Finally, durability under operational stresses including cyclic mechanical loading, thermal cycling, and ultraviolet exposure remains an open research area [184]. Early studies suggest that MXene-reinforced composites can maintain interfacial integrity under fatigue loading, but systematic lifetime data under coupled environmental and mechanical loading conditions remain limited [132]. UV-induced photo-oxidation, while useful for patterning and localized modification, may necessitate UV-blocking coatings for outdoor or long-term applications.

Standardized accelerated aging protocols for MXenes are not yet fully established; however, commonly employed approaches include elevated temperature/humidity exposure (e.g., 60–85 °C and >75% relative humidity), continuous air or oxygen flow environments, and UV-assisted oxidation testing. These methods provide comparative stability trends but lack universally accepted lifetime prediction models. Emerging efforts utilize Arrhenius-type scaling and diffusion-reaction modeling to estimate long-term degradation behavior, though these models remain material- and condition-specific. UV-induced photo-oxidation, while useful for patterning and localized modification, may necessitate UV-blocking coatings for outdoor or long-term applications [87,178,185,186].

Overall, improving the environmental robustness and durability of MXenes is essential for their transition from laboratory materials to reliable engineering components.

### 7.2. Scaling up Production and Cost Reduction

Although MXenes have been extensively studied at the laboratory level, large-scale production remains a significant challenge. Most reported synthesis routes currently operate at laboratory to small pilot scale, and translating these methods into economically viable, high-throughput manufacturing will require addressing chemical safety, process efficiency, material consistency, and supply chain constraints [187].

A major barrier to scale-up is the reliance of traditional MXene synthesis on hazardous fluoride-based etchants, particularly hydrofluoric acid (HF) [187]. Beyond safety concerns, HF handling increases regulatory burden, infrastructure costs, and waste management complexity [3]. As a result, substantial effort has been devoted to developing HF-free or reduced-HF etching routes, including electrochemical etching, molten salt methods, and alternative fluoride salt systems [3]. Among these, molten salt etching is especially attractive for scale-up due to its compatibility with large-batch reactors and reduced liquid waste streams [3]. However, these methods must still be optimized for continuous operation, high yield, and reproducibility before industrial adoption.

Process inefficiency further limits throughput and scalability. Conventional batch-based wet-etching and delamination routes typically produce low-solids MXene dispersions, requiring extensive washing, centrifugation, and solvent handling. These steps increase processing time, reduce yield, and complicate scale-up [188].

Recent work (2025–2026) confirms that this batch-processing paradigm represents a primary bottleneck to MXene industrialization, motivating a shift toward continuous-flow synthesis and high-concentration product formats. For example, a semi-continuous, 3D-printed flow reactor constructed from chemically inert PVDF was demonstrated in 2025 for the steady-state production of Ti_3_C_2_T_x_ [189]. Compared to conventional 24–48 h batch processes, this approach reduced manual handling of hazardous etchants, improved process monitoring, and enabled more consistent material output, a key requirement for industrial manufacturing [189].

In parallel, researchers have reported high-concentration MXene dispersions and “MXene dough” formulations (approaching ~30 mg·mL^−1^), which dramatically reduce solvent volume and downstream separation requirements [190]. These dense formats not only improve processing efficiency but also enhance shelf life by limiting dissolved oxygen access to MXene surfaces [190]. Together, these advances suggest that scalable MXene production will likely rely on continuous-flow reactors coupled with high-solids product handling, rather than traditional dilute batch synthesis routes.

Ensuring material consistency is another critical challenge. MXene properties depend sensitively on flake size, thickness, defect density, and surface termination chemistry, all of which can vary between batches [6]. For reliable engineering adoption, standardized material specifications will be required, analogous to grading systems used for carbon fibers or graphene.

The cost and availability of MAX phase precursors also play a central role in MXene economics. Producing high-purity MAX phases is energy and resource-intensive, and improvements in MAX synthesis efficiency, scale, and compositional tailoring could significantly reduce overall MXene costs [191]. Longer-term research directions include alternative synthesis pathways that bypass conventional MAX phases altogether, though such approaches remain at an early stage [192].

From an equipment standpoint, MXene manufacturing would benefit from reactor designs tailored for scalable chemical processing, such as modular electrochemical cells or large-volume molten salt reactors. Importantly, many of the unit operations involved (stirred reactors, filtration, electrolysis, and solvent recovery) are already standard in chemical engineering, suggesting that MXene scale-up is primarily a matter of process adaptation rather than entirely new infrastructure.

Finally, waste handling and recycling must be addressed for sustainable production. Fluoride-containing waste streams require careful management [193]. Strategies such as reagent recycling, closed-loop processing, and conversion of byproducts into reusable intermediates will be essential for reducing environmental impact and operating costs.

Overall, while significant obstacles remain, the path to scalable and cost-effective MXene production appears feasible through continued advances in chemistry, process engineering, and standardization.

### 7.3. Integration with Traditional Materials

For MXenes to transition from laboratory materials to engineering components, they must be successfully integrated into existing material systems, including polymers, metals, ceramics, and textiles. While MXenes offer exceptional multifunctionality, their 2D morphology, surface chemistry, and thermal sensitivity introduce unique integration challenges. However, recent progress in processing and interface engineering suggests viable pathways toward practical adoption.

#### 7.3.1. Polymer Integration

Polymers represent the most mature integration route for MXenes. Achieving uniform dispersion in thermoplastics and thermosets remains challenging due to high melt viscosity and the tendency of MXene sheets to restack [177]. Nonetheless, surface functionalization strategies such as grafting polymer chains onto MXene surfaces or using silane coupling agents have proven effective in improving compatibility and interfacial bonding [194]. These approaches enable MXenes to behave more like macromolecular fillers, enhancing load transfer, electrical percolation, and long-term stability within polymer networks [195].

#### 7.3.2. Metal Integration

Incorporating MXenes into metallic matrices is more complex due to MXene instability at elevated temperatures. Conventional melt processing is generally unsuitable, as MXenes readily oxidize or decompose [95]. To address this, researchers have explored low-temperature consolidation routes and are increasingly adopting spark plasma sintering (SPS) as a preferred consolidation method [196]. In Cu-MXene composites produced via SPS, partial retention of MXene-derived layered structures has been observed, leading to improved electrical conductivity, wear resistance, or interfacial strengthening [196]. Although some transformation to carbides or oxides is often unavoidable, these transformed phases can still contribute beneficially to reinforcement or interface modification [196].

#### 7.3.3. Ceramic Integration

Ceramic matrices present even greater challenges due to their high sintering temperatures. Direct retention of pristine MXenes is generally not feasible; however, MXenes can still play useful roles as sacrificial templates, interfacial modifiers, or layered reinforcements [138,197]. Low-temperature pressure-assisted sintering methods, such as SPS or cold sintering, have enabled the incorporation of MXenes or MXene-derived phases into ceramic systems [138,196]. Additionally, layered ceramic-MXene architectures inspired by nacre (“brick-and-mortar” structures) have demonstrated significantly enhanced fracture toughness through crack deflection and energy dissipation mechanisms, even when partial MXene transformation occurs [130].

#### 7.3.4. Textile and Flexible Substrate Integration

For wearable and flexible electronics, MXenes can be readily integrated with textiles and polymer films through solution-based processing [184]. Dip-coating, spray-coating, or printing MXene inks onto fibers produces conductive and sensing textiles with minimal processing complexity [184]. Adhesion is typically achieved via van der Waals interactions and hydrogen bonding, and durability can be improved through secondary polymer overcoats (e.g., polyurethane) [184]. While wash durability and abrasion resistance remain active challenges, recent studies demonstrate promising stability over repeated deformation and moderate laundering cycles [149,184].

#### 7.3.5. Biocompatibility and Biomedical Composites

When MXenes are considered for biomedical reinforcement or sensing applications, biocompatibility becomes critical. Current evidence suggests that MXene cytotoxicity depends strongly on surface chemistry, oxidation state, and dose [198]. Encapsulation within biopolymers such as chitosan, gelatin, or PEG has been shown to mitigate adverse biological responses while preserving MXene conductivity and mechanical reinforcement [179,198]. Long-term in vivo studies remain limited, but early results indicate potential for MXene-enabled scaffolds and implantable sensors.

#### 7.3.6. Standardization and Materials Qualification

A key barrier to industrial integration is the lack of standardized MXene specifications. Properties such as flake size distribution, surface termination chemistry, oxidation state, and electrical conductivity vary widely between laboratories [6]. Efforts are underway to establish consistent characterization protocols and performance benchmarks, which will be essential for defining application-specific MXene grades (e.g., “conductive grade” versus “structural reinforcement grade”) [199]. Engagement with standards of organizations such as ASTM and ISO is expected to play a critical role in this transition.

#### 7.3.7. Outlook

Looking forward, integration strategies are likely to favor hybrid architectures that combine MXenes with established materials rather than replacing them outright. Examples include carbon fiber composites with MXene-modified interphases, co-printed polymer-MXene structures for embedded sensing, and multifunctional fibers that combine mechanical strength with electrical or thermal functionality. While long-term stability, cost reduction, and manufacturing compatibility remain central challenges, the rapid pace of progress suggests that MXenes are well-positioned to enter niche, high-performance applications within the next decade.

Overall, MXenes occupy a compelling middle ground between structural fillers and functional additives. Their successful integration with traditional materials will depend not on eliminating their limitations, but on engineering around them; an approach increasingly supported by advances in processing, surface chemistry, and composite design. Table 6 summarizes the primary barriers to commercialization and the evolving strategies to overcome them.

## 8. Conclusions

MXenes represent a rapidly evolving class of two-dimensional transition metal carbides and nitrides whose unique structure and property relationships place them at the intersection of materials science and mechanical engineering. This work has reviewed the foundational aspects of MXenes, beginning with their layered crystal structure, tunable surface terminations, and compositional versatility, which collectively underpin their mechanical, electrical, and electrochemical behavior. The structural adaptability of MXenes combined with their high aspect ratios and strong in-plane bonding distinguishes them from conventional layered materials and enables property sets that are difficult to achieve in traditional engineering materials.

Advances in synthesis and processing have been central to translating MXenes from laboratory curiosities to mechanically relevant materials. While early batch-based wet etching routes constrained scalability, recent progress toward continuous-flow synthesis, high-concentration dispersions, and improved delamination efficiency marks a significant step toward industrial viability [189,190,193]. These developments are particularly important for mechanical engineers, as processing routes directly influence material uniformity, defect density, form factor, and integration into load-bearing or multifunctional systems. At the same time, synthesis challenges, such as oxidation sensitivity, surface termination control, and process safety, remain critical considerations for large-scale deployment.

The mechanical properties of MXenes further reinforce their relevance to mechanical engineering applications. High elastic moduli, strength retention at small thicknesses, and favorable interfacial bonding enable MXenes to function not only as standalone films but also as reinforcing agents in composites and layered architectures. When combined with polymers, metals, or ceramics, MXenes offer pathways to engineer stiffness, toughness, damping, and multifunctionality simultaneously. These attributes position MXenes as enabling materials rather than direct replacements for traditional structural materials.

Beyond purely mechanical behavior, the functional properties of MXenes, particularly their electrical conductivity, electrochemical activity, and surface-driven interactions, expand the design space for mechanically engineered systems. Unlike many conductive fillers, MXenes provide high conductivity at low loading fractions while maintaining processability and mechanical compliance. This combination is especially attractive for applications requiring structural integrity alongside sensing, actuation, thermal management, or energy interaction, all of which are increasingly relevant in modern mechanical systems.

Applications in mechanical engineering highlight MXenes’ potential to contribute to next-generation technologies, from multifunctional composites and coatings to smart structures and mechanically robust energy systems. Importantly, these applications leverage MXenes not as isolated materials, but as integrated components within engineered systems, aligning with the systems-level thinking central to mechanical engineering practice.

Despite this progress, several challenges continue to shape the future trajectory of MXenes. Scalable manufacturing, long-term environmental stability, standardized characterization methods, and cost-effective processing remain key barriers to widespread adoption [125,190,191]. Addressing these challenges will require interdisciplinary collaboration, particularly between materials scientists and mechanical engineers, to ensure that processing innovations align with performance requirements and manufacturing constraints.

In summary, MXenes have progressed from emerging two-dimensional materials to mechanically relevant building blocks with significant future potential. While they are not yet ready to supplant conventional engineering materials, their unique combination of mechanical robustness and functional versatility positions them as powerful enablers of multifunctional mechanical systems. As synthesis methods mature and integration strategies improve, MXenes are poised to play an increasingly influential role in the design of advanced materials and structures for mechanical engineering applications.

## Figures and Tables

**Table 1:** Comparison of compositional features across major MXene structural classes.

MXene Structure Type	Transition Metal (M) Components	Carbon/Nitrogen Component (X)	Surface Terminations (T_x_)	References
Single Transition-Metal	1	1 (C or N)	1 to 3+ (Mixed)	[7]
In-Plane Ordered Double Transition Metal	1 or 2	1	1 to 3+	[8]
Out-Plane Ordered Double Transition Metal	2	1	1 to 3+	[8]
Solid-Solution (M-site)	2+ (Randomly Distributed)	1	1 to 3+	[12]
Solid-Solution (X-site)	1	2 (C and N)	1 to 3+	[12]
High-Entropy	5 or more	1 or 2	1 to 3+	[12]

**Table 2:** Advantages and Disadvantages of MXene Synthesis Methods.

Synthesis Approaches			
Synthesis Method	Advantages	Disadvantages	Reference
Top-Down			
HCL Electrochemical Etching	Synergistic potential when combined with molten salts for optimized yields.	Risk of over-etching, which can degrade the structural integrity of the MXene. Larger flake sizes (>2 μm) Very low yield (3–5%)	[27,31]
Lewis Acid Molten Salt Etching	Highly scalable for industrial applications. Enables synthesis of unique MXene chemistries inaccessible via HF-based methods. Smaller flake sizes (as small as 1 μm)	High interlayer bonding makes subsequent delamination into single layers difficult. Lower yield (60–80%)	[27,31]
Halogen-Based Etching	Produces MXenes with superior electrical conductivity compared to traditional HF methods. Smaller flake sizes (as low as 0.5 μm) Higher yield (70–90%)	Requires stringent safety protocols and specialized handling equipment.	[3,32]
Microwave Synthesis	Precise, directed heating leads to high purity. Significantly reduced reaction times compared to oven heating. High yield (70–90%)	Limited scalability due to restricted penetration depth and sample size. Very large flake size (35–45 μm)	[28,33]
Hot Pressing	Produces high-density bulk materials.	Time-intensive processing cycles. Generally unsuitable for large-format manufacturing. Lower yield (10–15%)	[28,34]
Hot Isostatic Pressing	Achieves superior material density and purity via uniform pressure application.	Prolonged processing times. Inefficient for large-scale throughput. Lower yield (10–15%)	[28,34]
Spark Plasma Sintering	Extremely rapid (minutes vs. hours). Maintains low grain growth and high density. High yield (94.42%)	Scalability is limited by the requirement for specific conductive molds.	[28,35]
Pressureless Sintering	Yields highly homogeneous material distributions.	Lengthy baking durations. Risk of thermal instability or ”thermal explosion” during the heating cycle. Larger flake sizes (3 μm)	[28,36]
Bottom-Up			
Physical Vapor Deposition	Exceptional control over purity and density at the atomic level.	Restricted by “line-of-sight” requirements for laser or evaporation sources.	[28]

**Table 3:** Electrochemical performance metrics for Ti_3_C_2_T_x_ MXenes under different electrolytes and current densities. Reproduced from [44], Nanomaterials, MDPI, Open access.

Electrode	Electrolyte	Scan Rate/Current Density	Initial Capacitance (IC)	Cycle Number (CN)	Capacity After Cycles (AC)
Ti_3_C_2_T_x_	1 M KOH	1 Ag^−1^	350 Fcm^−3^	10,000	~94%
Ti_3_C_2_T_x_	1 M H_2_SO_4_	5 Ag^−1^	415 Fcm^−3^	10,000	~100%
Ti_3_C_2_T_x_	1 M H_2_SO_4_	10 Ag^−1^	900 Fcm^−3^	10,000	~100%
Ti_3_C_2_T_x_	1 M H_2_SO_4_	10 Ag^−1^	499 Fg^−1^	10,000	~100%
Ti_3_C_2_T_x_	6 M KOH	5 Ag^−1^	188 Fg^−1^	5000	~100%
Ti_3_C_2_T_x_	1 M H_2_SO_4_	5 Ag^−1^	215 Fg^−1^	10,000	~100%
Ti_3_C_2_T_x_	1 M H_2_SO_4_	5 Ag^−1^	892 Fg^−1^	10,000	~100%

**Table 4:** Intrinsic monolayer (graphene, MoS_2_, h-BN) vs. representative single-layer/flake Ti_3_C_2_T_x_.

Property	Ti_3_C_2_T_x_ MXene	Pristine Graphene	Pristine MoS_2_	Pristine h-BN	Source
Young’s Modulus [GPa]	300–360 (AFM nanoindentation; suspended flakes	1000 (AFM nanoindentation; suspended monolayer)	270 ± 100 (AFM nanoindentation)	865 ± 73 (AFM nanoindentation)	[41,48,49,50]
Fracture Strength [GPa]	15.4 (AFM nanoindentation; high variability due to defects/terminations)	120–140 (AFM nanoindentation)	16–30 (AFM nanoindentation)	65–75 (AFM nanoindentation)	[41,48,49,50]
In-Plane Electrical Conductivity [S·m^−1^]	1.1×10^6^(monolayer FET devices; upper-bound)	1.46 ± 0.82 × 10^6^ (C-AFM)	Wide-bandgap semiconductor (negligible intrinsic conductivity)	Wide-bandgap insulator (extremely low leakage current)	[51,52,53,54]
Thermal Conductivity (κ) [W·m^−1^·K^−1^]	~2.3 (in-plane, Raman thermometry; validated by laser flash analysis)	2000–5000 (Raman optothermal; suspended)	30.5–38.5 (Raman thermometry; monolayer)	200–400 (theoretical upper bound; experimental typically lower)	[54,55,56,57]
Thickness per Monolayer [nm]	0.98–1.8 (AFM and Raman spectroscopy; includes surface terminations/absorbates)	0.4 to 1.7 (AFM and Raman Spectroscopy	0.65 to 0.72 (AFM and Raman Spectroscopy)	0.44 to 0.6 (AFM and Raman Spectroscopy)	[48,58,59,60]
Monolayer/Device-Grade Cost	$4000–8000/g (colloidal monolayer dispersion; not available as continuous cm^2^ films))	$500–1000/cm^2^ (mechanical exfoliation or CVD films)	$300–600/cm^2^ (CVD monolayer films)	$400–700/cm^2^ (CVD monolayer films)	[61,62,63,64]
Bulk/Scalable Cost [$/g]	$12.20–20.33 (MAX phase synthesis + etching + delamination)	$1–100 (graphite-derived; quality dependent)	$0.5–5 (crystalline powder/bulk molybdenite-derived, quality dependent)	$1–10 (powder/fully synthetic ceramic, quality dependent)	[65,66]
Environmental Stability	Low (oxidation in ambient conditions; hours–days depending on termination and storage	High (chemically stable; slow oxidation over long timescales)	Moderate (oxidizes at elevated temperature/defects)	Very high (chemically inert, oxidation-resistant)	[67,68]

**Table 5:** Representative MXene applications in mechanical engineering, with examples of material systems, key functions, and performance benefits. (TRL: Technology Readiness Level.)

Application Category	Material System	Key Function	Performance Benefit	TRL	References
Structural Composites	Ti_3_C_2_T_x_ MXene/Epoxy	Mechanical reinforcement, load transfer	Tensile strength increase ~51%; enhanced modulus & toughness	(Lab scale)	[121]
Energy & Flexible Devices	Ti_3_C_2_T_x_ MXene film electrode	Conductive, flexible current collector	>350 F·g^−1^ capacitance; ~90% retention after 10 k cycles	(Prototype)	[122,123]
Sensing & Actuation	MXene/PDMS Strain Sensor	Piezoresistive strain detection	Detects <0.2% strain; stable operation >5 k cycles	(Lab prototype)	[124]
Thermal Management	MXene/Graphene composite film	Heat spreading, EMI shielding	In-plane thermal cond. ~26–37 W·m^−1^·K^−1^; robust cycling stability	(Lab/Pilot)	[125]
Protective Coatings	MXene/Polymer coating	EMI shielding & corrosion barrier	24–92 dB EMI shielding; ~99.2% Inhibition Efficiency	(Early prototype)	[126,127]
Separation Membranes	Ti_3_C_2_T_x_/GO laminate	Ion sieving (desalination)	High salt ion selectivity; up to 10,000× increased water permeability (Compared to GO-based and commercial membranes)	(Pilot testing)	[128]

**Table 6:** Key Challenges and Research Directions for MXene Commercialization.

Challenge	Impact	Current Mitigation Strategies	Emerging Research Directions	References
Oxidation & Environmental Instability	Degraded conductivity, shortened device lifetime	Polymer encapsulation, inert storage, controlled-atmosphere processing	ALD coatings, intrinsic doping (B, Si), cryogenic preservation, DFT-guided termination design	[179,181,200,201]
Scalable & Sustainable Synthesis	High cost, low yield, hazardous waste	Fluoride-free etching (electrochemical, molten salt), solvent recycling	Continuous-flow reactors, closed-loop systems, supercritical CO_2_ exfoliation	[109,189,193]
Interfacial Integration	Poor dispersion, weak bonding, delamination in composites	Surface functionalization, surfactant-assisted dispersion, ultrasonic processing	Hybrid 2D/3D architectures, cold-spray deposition, in situ polymerization	[133,144,199]
Lack of Standardization	Inconsistent material quality, unreliable device performance	Lab-specific characterization protocols	International standards for flake size, purity, conductivity, and oxidation state	[76,190,200]
Biocompatibility & Safety	Limited use in biomedical and consumer applications	Biopolymer encapsulation (chitosan, gelatin), hydrogel blending	Engineered degradable scaffolds, long-term in vivo stability studies, and surface passivation	[179,197,198]

## Data Availability

No new data were created or analyzed in this study. Data sharing is not applicable to this article.

## References

[1] Protyai M.I.H., Rashid A.B. (2024). A Comprehensive Overview of Recent Progress in MXene-Based Polymer Composites: Their Fabrication Processes, Advanced Applications, and Prospects. Heliyon.

[2] Lamiel C., Hussain I., Warner J.H., Zhang K. (2023). Beyond Ti-Based MXenes: A Review of Emerging Non-Ti Based Metal-MXene Structure, Properties, and Applications. Mater. Today.

[3] Antony Jose S., Price J., Lopez J., Perez-Perez E., Menezes P.L. (2025). Advances in MXene Materials: Fabrication, Properties, and Applications. Materials.

[4] Saleth L.R., Gupta M., Sharma G., Verma E., Dhingra S. (2025). Meta-Analyses of the Evolution of MXene Synthesis for Bioengineering and Artificial Intelligence-Driven Applications. Commun. Mater..

[5] Gautam R., Marriwala N., Devi R. (2023). A Review: Study of Mxene and Graphene Together. Meas. Sens..

[6] Bao W., Shen H., Zeng G., Zhang Y., Wang Y., Cui D., Xia J., Jing K., Liu H., Guo C. (2025). Engineering the next Generation of MXenes: Challenges and Strategies for Scalable Production and Enhanced Performance. Nanoscale.

[7] Khan I.A., Yogarathinam L.T., Younas H., Mushtaq A., Aljundi I.H., Baig N. (2026). The Rise of MXenes: Synthesis, Properties, and Fabrication of Advanced Membranes. Desalination.

[8] Anasori B., Xie Y., Beidaghi M., Lu J., Hosler B.C., Hultman L., Kent P.R.C., Gogotsi Y., Barsoum M.W. (2015). Two-Dimensional, Ordered, Double Transition Metals Carbides (MXenes). ACS Nano.

[9] Jiang J., Bai S., Zou J., Liu S., Hsu J.-P., Li N., Zhu G., Zhuang Z., Kang Q., Zhang Y. (2022). Improving Stability of MXenes. Nano Res..

[10] Rostami M., Badiei A. (2024). Molten Salt-Shielded Preparation of the Ultrathin Nanosheets of Ti_3_C_2_Cl_2_ @TiO_2_ Coupled with Fe_3_O_4_@C-C_3_N_4_ for Assessing Photocatalytic Activity. Ceram. Int..

[11] Anayee M., Wang R.J., Downes M., Ippolito S., Gogotsi Y. (2025). Layer-by-Layer Mechanism of the MAX Phase to MXene Transformation. Matter.

[12] Anasori B., Naguib M. (2023). Guest Editors Two-Dimensional MXenes. MRS Bull..

[13] Magnuson M., Halim J., Näslund L.-Å. (2018). Chemical Bonding in Carbide MXene Nanosheets. J. Electron Spectrosc. Relat. Phenom..

[14] Shekhirev M., Shuck C.E., Sarycheva A., Gogotsi Y. (2021). Characterization of MXenes at Every Step, from Their Precursors to Single Flakes and Assembled Films. Prog. Mater. Sci..

[15] Wicklein B., Valurouthu G., Yoon H., Yoo H., Ponnan S., Mahato M., Kim J., Ali S.S., Park J.Y., Gogotsi Y. (2024). Influence of MXene Composition on Triboelectricity of MXene-Alginate Nanocomposites. ACS Appl. Mater. Interfaces.

[16] Thangavelu H.H.S., Huang C., Chabanais F., Palisaitis J., Persson P.O.Å. (2026). A Review on MXene Terminations. Adv. Funct. Mater..

[17] Rems E., Hu Y.-J., Gogotsi Y., Dominko R. (2024). Pivotal Role of Surface Terminations in MXene Thermodynamic Stability. Chem. Mater..

[18] Liu J., Yu W., Zhao Z., Liu D., Liu S., Wang J., Ma M., Yu Q., Yang N. (2023). 3D Honeycomb Fe/MXene Derived from Prussian Blue Microcubes with a Tunable Structure for Efficient Low-Frequency and Flexible Electromagnetic Absorbers. ACS Appl. Mater. Interfaces.

[19] Liu Z., Cui Y., Li Q., Zhang Q., Zhang B. (2022). Fabrication of Folded MXene/MoS_2_ Composite Microspheres with Optimal Composition and Their Microwave Absorbing Properties. J. Colloid Interface Sci..

[20] Venkateshalu S., Shariq M., Kim B., Patel M., Mahabari K.S., Choi S.-I., Chaudhari N.K., Grace A.N., Lee K. (2023). Recent Advances in MXenes: Beyond Ti-Only Systems. J. Mater. Chem. A.

[21] Chuanfang Z. (2019). Two-Dimensional Transition Metal Carbides and Nitrides (MXenes): Synthesis, Properties, and Electrochemical Energy Storage Applications. Energy Environ. Mater..

[22] Lim K.R.G., Shekhirev M., Wyatt B.C., Anasori B., Gogotsi Y., Seh Z.W. (2022). Fundamentals of MXene Synthesis. Nat. Synth..

[23] Yongbin W. (2024). Ultrafast Synthesis of MXenes in Minutes via Low-Temperature Molten Salt Etching. Adv. Mater..

[24] Jin S., Guo Y., Wang F., Zhou A. (2023). The Synthesis of MXenes. MRS Bull..

[25] Chen J., Jin Q., Li Y., Shao H., Liu P., Liu Y., Taberna P.-L., Huang Q., Lin Z., Simon P. (2023). Molten Salt-Shielded Synthesis (MS3) of MXenes in Air. ENERGY Environ. Mater..

[26] Maleski K., Alhabeb M., Anasori B., Gogotsi Y. (2019). Top-Down MXene Synthesis (Selective Etching). 2D Metal Carbides and Nitrides (MXenes): Structure, Properties and Applications.

[27] Wong A.J.Y., Lim K.R.G., Seh Z.W. (2022). Fluoride-Free Synthesis and Long-Term Stabilization of MXenes. J. Mater. Res..

[28] Gonzalez-Julian J. (2021). Processing of MAX Phases: From Synthesis to Applications. J. Am. Ceram. Soc..

[29] Mishra R.K., Sarkar J., Verma K., Chianella I., Goel S., Nezhad H.Y. (2024). Exploring Transformative and Multifunctional Potential of MXenes in 2D Materials for Next-Generation Technology. Open Ceram..

[30] Majid A., Jabeen A. (2023). Synthesis and Properties of Layered Materials. Layeredness in Materials.

[31] Majid A., Jabeen A. (2023). Characteristics, Strategies and Applications of Layered Materials: An Introduction. Layeredness in Materials.

[32] Akinay Y., Topuz M., Karatas E., Gokdemir M.E., Cetin T., Polat S., Abaszade R., Singh M., Imanov H. (2026). Fundamentals of MXene Synthesis Steps and Their Characterization Techniques: Morphological, Structural, and Electrochemical Properties. ChemElectroChem.

[33] Paskaš J., Dujović M., Govedarica M., Radonić V., Srdić V.V., Kanas N. (2026). Ultrafast Microwave Synthesis of Ti-Based MXenes with High Yields. RSC Adv..

[34] Shahin S.M.T., Montazer M. (2026). A Comprehensive Review on Synthesis and Application of MAX/MXenes as 2D Nanoreactants with a Glance at the Modification of Materials. Results Eng..

[35] Shichalin O.O., Ivanov N.P., Seroshtan A.I., Nadaraia K.V., Simonenko T.L., Gurin M.S., Papynov E.K. (2024). Spark Plasma Sintering of Ti2AlC/TiC MAX-Phase Based Composite Ceramic Materials and Study of Their Electrochemical Characteristics. Ceram. Int..

[36] Bi L., Perry W., Wang R.J., Lord R., Hryhorchuk T., Inman A., Gogotsi O., Balitskiy V., Zahorodna V., Baginskiy I. (2024). MXene Functionalized Kevlar Yarn via Automated, Continuous Dip Coating. Adv. Funct. Mater..

[37] Tian S., Zhou K., Huang C.-Q., Qian C., Gao Z., Liu Y. (2022). Investigation and Understanding of the Mechanical Properties of MXene by High-Throughput Computations and Interpretable Machine Learning. Extrem. Mech. Lett..

[38] Pešić I., Petrović M., Vuksanović M., Popović M., Rabasović M.S., Šević D., Radojević V. (2022). Structural, Optical, and Mechanical Characterization of PMMA-MXene Composites Functionalized with MEMO Silane. Nanocomposites.

[39] Jayan R., Vashisth A., Islam M.M. (2022). First-principles Investigation of Elastic and Electronic Properties of Double Transition Metal Carbide MXenes. J. Am. Ceram. Soc..

[40] Cheng Y., Xie Y., Cao H., Li L., Liu Z., Yan S., Ma Y., Liu W., Yue Y., Wang J. (2023). High-Strength MXene Sheets through Interlayer Hydrogen Bonding for Self-Healing Flexible Pressure Sensor. Chem. Eng. J..

[41] Rong C., Su T., Li Z., Chu T., Zhu M., Yan Y., Zhang B., Xuan F.-Z. (2024). Elastic Properties and Tensile Strength of 2D Ti_3_C_2_T_x_ MXene Monolayers. Nat. Commun..

[42] Zhou Q., Maithani V., Prati M., Ippolito S., Creighton M.A., Gogotsi Y., Mukherjee S., Cao C. (2025). Interfacial Shear Strength of MXene Interfaces. Cell Rep. Phys. Sci..

[43] Yang Q., Eder S.J., Martini A., Grützmacher P.G. (2023). Effect of Surface Termination on the Balance between Friction and Failure of Ti_3_C_2_T_x_ MXenes. npj Mater. Degrad..

[44] Ibrahim Y., Mohamed A., Abdelgawad A.M., Eid K., Abdullah A.M., Elzatahry A. (2020). The Recent Advances in the Mechanical Properties of Self-Standing Two-Dimensional MXene-Based Nanostructures: Deep Insights into the Supercapacitor. Nanomaterials.

[45] Khaledialidusti R. (2020). Temperature-Dependent Mechanical Properties of Ti_n+1_C_n_O_2_ (n = 1, 2) MXene Monolayers: A First-Principles Study. Phys. Chem. Chem. Phys..

[46] Adstedt K., Buxton M.L., Henderson L.C., Hayne D.J., Nepal D., Gogotsi Y., Tsukruk V.V. (2023). 2D Graphene Oxide and MXene Nanosheets at Carbon Fiber Surfaces. Carbon.

[47] Buxton M.L., Brackenridge J., Poliukhova V., Nepal D., Bunning T.J., Tsukruk V.V. (2025). Surface Mapping of Functionalized Two-Dimensional Nanosheets: Graphene Oxide and MXene Materials. Langmuir.

[48] Lee C., Wei X., Kysar J.W., Hone J. (2008). Measurement of the Elastic Properties and Intrinsic Strength of Monolayer Graphene. Science.

[49] Akhter M.J., Kuś W., Mrozek A., Burczyński T. (2020). Mechanical Properties of Monolayer MoS_2_ with Randomly Distributed Defects. Materials.

[50] Falin A., Cai Q., Santos E.J.G., Scullion D., Qian D., Zhang R., Yang Z., Huang S., Watanabe K., Taniguchi T. (2017). Mechanical Properties of Atomically Thin Boron Nitride and the Role of Interlayer Interactions. Nat. Commun..

[51] Jia L., Zhou S., Ahmed A., Yang Z., Liu S., Wang H., Li F., Zhang M., Zhang Y., Sun L. (2023). Tuning MXene Electrical Conductivity towards Multifunctionality. Chem. Eng. J..

[52] Lim S., Park H., Yamamoto G., Lee C., Suk J.W. (2021). Measurements of the Electrical Conductivity of Monolayer Graphene Flakes Using Conductive Atomic Force Microscopy. Nanomaterials.

[53] Li X., Mullen J.T., Jin Z., Borysenko K.M., Buongiorno Nardelli M., Kim K.W. (2013). Intrinsic Electrical Transport Properties of Monolayer Silicene and MoS_2_ from First Principles. Phys. Rev. B.

[54] Electrical Conductivity vs Thermal Conductivity in Hexagonal Boron Nitride. https://eureka.patsnap.com/report-research-on-electrical-conductivity-vs-thermal-conductivity-in-hexagonal-boron-nitride.

[55] Han Z., Ruan X. (2023). Thermal Conductivity of Monolayer Graphene: Convergent and Lower than Diamond. Phys. Rev. B.

[56] Yan R., Simpson J.R., Bertolazzi S., Brivio J., Watson M., Wu X., Kis A., Luo T., Hight Walker A.R., Xing H.G. (2014). Thermal Conductivity of Monolayer Molybdenum Disulfide Obtained from Temperature-Dependent Raman Spectroscopy. ACS Nano.

[57] Srivastava P., Rohini R. (2026). In Situ Characterization of Temperature-Dependent Thermal Conductivity in MXene (Ti_3_C_2_T_x_) Using Raman Spectroscopy. J. Phys. Chem. C.

[58] Huang Y., Spiece J., Parker T., Lee A., Gogotsi Y., Gehring P. (2024). Violation of the Wiedemann–Franz Law and Ultralow Thermal Conductivity of Ti_3_C_2_T_x_ MXene. ACS Nano.

[59] Li X., Zhu H. (2015). Two-Dimensional MoS_2_: Properties, Preparation, and Applications. J. Mater..

[60] Hexagonal Boron Nitride (h-BN) Structure and Band Gap. https://www.ossila.com/pages/hexagonal-boron-nitride-h-bn-structure.

[61] MSE PRO Ti_3_C_2_T_x_ MXene Multilayer Nanoflakes. https://www.msesupplies.com/products/mse-pro-ti3c2tx-mxene-multilayer-nanoflakes.

[62] Mechanically Exfoliated Single Crystal Graphene on SiO_2_/Si (SiO_2_: 90nm Thick). https://www.acsmaterial.com/mechanically-exfoliated-graphene-on-sio2-si-90nm.html?srsltid=AfmBOorNy3KdAqPLIXGKkthxd9ks9Ttsy-8lRvcXxDdTp4f0GXEFslZy.

[63] MoS_2_-Full Area Monolayer on c-Cut Sapphire. https://2dsemiconductors.com/mos2-full-area-monolayer-on-c-cut-sapphire/.

[64] H-BN CVD Transferred on SiO_2_/Si. https://2dsemiconductors.com/h-bn-cvd-transferred-on-sio2-si/.

[65] Zaed M.A., Tan K.H., Abdullah N., Saidur R., Pandey A.K., Saleque A.M. (2024). Cost Analysis of MXene for Low-Cost Production, and Pinpointing of Its Economic Footprint. Open Ceram..

[66] MilliporeSigma|Life Science Products & Service Solutions. https://www.sigmaaldrich.com/US/en.

[67] Huynh N.-P., Kim H.-J., Chung K.-H. (2025). Long-Term Wear Characteristics of Single-Layer h-BN, MoS_2_, and Graphene. Tribol. Int..

[68] Marquis E., Benini F., Anasori B., Rosenkranz A., Righi M.C. (2023). Effect of Vacancies and Edges in Promoting Water Chemisorption on Titanium-Based MXenes. Nano Converg..

[69] Priyadarshini S. (2023). Effect of Graphene and MXene as 2D Filler Material on Physico-Mechanical Properties of Hemp/E-Glass Fibers Reinforced Hybrid Composite: A Comparative Study. Polym. Compos..

[70] Pandey M., Deshmukh K., Raman A., Asok A., Appukuttan S., Suman G.R. (2024). Prospects of MXene and Graphene for Energy Storage and Conversion. Renew. Sustain. Energy Rev..

[71] Vaishag P.V., Noh J.-S. (2024). A Comparative Review of Graphene and MXene-Based Composites towards Gas Sensing. Molecules.

[72] Ren K., Zhang G., Zhang L., Qin H., Zhang G. (2023). Ultraflexible Two-Dimensional Janus Heterostructure Superlattice: A Novel Intrinsic Wrinkled Structure. Nanoscale.

[73] Huang L., Shu H., Wang G., Mu W., Li J., Ren K. (2025). Thermal Transport Characteristics of Wrinkle Interface in Ultraflexible MoSSe–WX2 (X = S, Se) Heterostructure. ACS Appl. Mater. Interfaces.

[74] Liu L., Song Z., Wang J., Shen L., Tu B., Wang S., Mao D. (2016). Synthesis of Fe_2_O_3_/Graphene Composite Anode Materials with Good Cycle Stability for Lithium-Ion Batteries. Int. J. Electrochem. Sci..

[75] Jahn Y.M., Alboteanu G., Mordehai D., Ya’akobovitz A. (2024). Strain Engineering of the Mechanical Properties of Two-Dimensional WS2. Nanoscale Adv..

[76] Stratulat A.-M., Nesterova V., Korostelev V., Beidaghi M., Mochalin V., Klyukin K. (2025). Defect-Driven Degradation of MXenes in Aqueous Environments and Mitigation Strategies: Insights from First-Principles. ACS Nano.

[77] Gouveia J.D., Gomes J.R.B. (2022). Structural and Energetic Properties of Vacancy Defects in MXene Surfaces. Phys. Rev. Mater..

[78] Qin L. (2026). Defect Engineering and Effect of Vacancy Concentration on the Electrochemical Performance of V-Based MXenes. Energy Environ. Mater..

[79] Ronchi R.M., Chen N., Halim J., Persson P.O.Å., Rosen J. (2025). Defect Engineering of Mo_2−x_CTz MXenes through Precursor Alloying and Effects on Electrochemical Properties. Chem. Mater..

[80] Ye L., Mei X., Tang Z., Liu B., Xu S., Zheng H., Wang J., Guan S. (2025). MXene Defect Engineering for Optimizing the Mechanical Properties of Ti_3_C_2_T_X_/ZK61 Composites. Mater. Sci. Eng. A.

[81] Gao J., Xuan X., Tang Y., Xie Y., Bi Z., Zou J., Li L., Yang C. (2025). Thermal Stability of MXene: Current Status and Future Perspectives. Small.

[82] Marquez K.P., Sisican K.M.D., Ibabao R.P., Malenab R.A.J., Judicpa M.A.N., Henderson L., Zhang J., Usman K.A.S., Razal J.M. (2024). Understanding the Chemical Degradation of Ti_3_C_2_T*_x_* MXene Dispersions: A Chronological Analysis. Small Sci..

[83] Doo S., Chae A., Kim D., Oh T., Ko T.Y., Kim S.J., Koh D.-Y., Koo C.M. (2021). Mechanism and Kinetics of Oxidation Reaction of Aqueous Ti_3_C_2_T_x_ Suspensions at Different pHs and Temperatures. ACS Appl. Mater. Interfaces.

[84] Li H., Wang L., Hou S., Xie J., Li Z., Hu Q., Zhou A., Liu X. (2024). Effect of Morphology and Structure of MXene Ti_3_C_2_T_x_ on Mechanical, Thermal Properties of PEEK Nanocomposite. Carbon.

[85] Wan C., Wang Y., Wang N., Norimatsu W., Kusunoki M., Koumoto K. (2010). Development of Novel Thermoelectric Materials by Reduction of Lattice Thermal Conductivity. Sci. Technol. Adv. Mater..

[86] Zhu M., Lu C., Liu L. (2023). Progress and Challenges of Emerging MXene Based Materials for Thermoelectric Applications. iScience.

[87] Han M., Zhang D., Shuck C.E., Gogotsi Y. (2021). Ultralow and Selective Infrared Emission from MXenes. arXiv.

[88] Hu X., Fan Q., Wang S., Chen Y., Wang D., Chen K., Ge F., Zhou W., Liang K. (2024). Two-Dimensional MXenes: Innovative Materials for Efficient Thermal Management and Safety Solutions. Research.

[89] Liu Y., Zou W., Zhao N., Xu J. (2023). Electrically Insulating PBO/MXene Film with Superior Thermal Conductivity, Mechanical Properties, Thermal Stability, and Flame Retardancy. Nat. Commun..

[90] Chen L., Shi X., Yu N., Zhang X., Du X., Lin J. (2018). Measurement and Analysis of Thermal Conductivity of Ti_3_C_2_T_x_ MXene Films. Materials.

[91] Li X., Wang H., Li H., Chen Y., Ni Y., Xia Y. (2023). Zr_3_C_2_O_2_ MXene as Promising Candidate for NH_3_ Sensor with High Sensitivity and Selectivity at Room Temperature. Appl. Surf. Sci..

[92] FitzPatrick J., Gogotsi Y. (2025). MXene Polymer Composites: A Review. MRS Bull..

[93] Lee S., Kim J. (2021). Incorporating MXene into Boron Nitride/Poly(Vinyl Alcohol) Composite Films to Enhance Thermal and Mechanical Properties. Polymers.

[94] Kang R., Zhang Z., Guo L., Cui J., Chen Y., Hou X., Wang B., Lin C.-T., Jiang N., Yu J. (2019). Enhanced Thermal Conductivity of Epoxy Composites Filled with 2D Transition Metal Carbides (MXenes) with Ultralow Loading. Sci. Rep..

[95] Wyatt B.C., Nemani S.K., Desai K., Kaur H., Zhang B., Anasori B. (2021). High-Temperature Stability and Phase Transformations of Titanium Carbide (Ti_3_C_2_T*_x_*) MXene. J. Phys. Condens. Matter.

[96] Fu J., Lian W., Deng Y., Fang Z., Cheng Q. (2025). Isotropic Thermal Insulating Cuttlebone-Inspired MXene Aerogel. Natl. Sci. Rev..

[97] Wang X., Hu X., Liu Z., Zhu C., Shen R., Quan B., Yan X., Wang W., Lu X., Qu J. (2025). Interpenetrating Double-Network ANF/MXene-K+ Aerogels Enable Integrated Electromagnetic Interference Shielding, Infrared Camouflage, and Joule Heating in Adaptive Multifunctional Systems. Nano Res..

[98] Auh Y.H., Neal N.N., Arole K., Regis N.A., Nguyen T., Ogawa S., Tsuda Y., Yoshigoe A., Radovic M., Green M.J. (2025). Nacre-like MXene/Polyacrylic Acid Layer-by-Layer Multilayers as Hydrogen Gas Barriers. ACS Appl. Mater. Interfaces.

[99] Zhang C.J., Pinilla S., McEvoy N., Cullen C.P., Anasori B., Long E., Park S.-H., Seral-Ascaso A., Shmeliov A., Krishnan D. (2017). Oxidation Stability of Colloidal Two-Dimensional Titanium Carbides (MXenes). Chem. Mater..

[100] Cheng L., Liu G., Zhao J., Jin W. (2021). Two-Dimensional-Material Membranes: Manipulating the Transport Pathway for Molecular Separation. Acc. Mater. Res..

[101] Wang X., Li X., Cui L., Liu Y., Fan S. (2022). Improvement of Gas Barrier Properties for Biodegradable Poly(Butylene Adipate-Co-Terephthalate) Nanocomposites with MXene Nanosheets via Biaxial Stretching. Polymers.

[102] Dutta A., Dutta G. (2016). Comparing Optimum Barrier Variables of Aluminium and MPET Foil Based Laminates for Coffee Packaging. J. Appl. Poult. Res..

[103] Helal M.I., Sinopoli A., Gladich I., Tong Y., Alfahel R., Gomez T., Mahmoud K.A. (2024). Understanding the Swelling Behavior of Ti_3_C_2_T_x_ MXene Membranes in Aqueous Media. J. Mater. Chem. A.

[104] Ajith S., Almomani F., Qiblawey H. (2024). Emerging 2D MXene-Based Polymeric Membranes for Water Treatment and Desalination. J. Environ. Chem. Eng..

[105] Liu J.-J., Yang W.-J., Xu Y., Yuen A.C.Y., Chen T.B.Y., Wei C.-X., Zhu S.-E., Yeoh G.-H., Yang W., Lu H.-D. (2022). MXene-Based Films via Scalable Fabrication with Improved Mechanical and Antioxidant Properties for Electromagnetic Interference Shielding. Compos. Commun..

[106] Zhang B., Wong P.W., Guo J., Zhou Y., Wang Y., Sun J., Jiang M., Wang Z., An A.K. (2022). Transforming Ti_3_C_2_T_x_ MXene’s Intrinsic Hydrophilicity into Superhydrophobicity for Efficient Photothermal Membrane Desalination. Nat. Commun..

[107] Wan H., Min W., Li J., Li Y., Chen C. (2026). Design and Performance Study of Hydrogen-Resistant Coatings Based on MOF’s Porous Filled MXene Layered Structure. Corros. Sci..

[108] Qiang Y., Ran B., Li M., Xu Q., Peng J. (2023). GO-Functionalized MXene towards Superior Anti-Corrosion Coating. J. Colloid Interface Sci..

[109] Nazarlou Z., Peighambardoust N.S., Motlagh P.L., Aydemir U. (2025). Enhancing Corrosion Resistance and Tribomechanical Characteristics of Powder Coatings via the Integration of Functionalized HF-Free MXene Reinforcements. Adv. Mater. Interfaces.

[110] Lu X., Gu X., Shi Y. (2023). A Review on the Synthesis of MXenes and Their Lubrication Performance and Mechanisms. Tribol. Int..

[111] Sui X., Liu J., Zhao G., Wang X., Han Y., Gachot C. Ti_3_C_2_T_x_ MXene Modified Alkyl Imidazolium Ionic Liquid-Stearic Acid Composite Materials as a Potential Lubricant for Steel/Steel Contact. Wear.

[112] Macknojia A.Z., Ayyagari A., Shevchenko E., Berman D. (2023). MXene/Graphene Oxide Nanocomposites for Friction and Wear Reduction of Rough Steel Surfaces. Sci. Rep..

[113] Nguyen H.T., Chung K.-H. (2020). Assessment of Tribological Properties of Ti_3_C_2_ as a Water-Based Lubricant Additive. Materials.

[114] Li Y., Huang S., Wei C., Zhou D., Li B., Mochalin V.N., Wu C. (2022). Friction between MXenes and Other Two-Dimensional Materials at the Nanoscale. Carbon.

[115] Rosenkranz A., Righi M.C., Sumant A.V., Anasori B., Mochalin V.N. (2023). Perspectives of 2D MXene Tribology. Adv. Mater..

[116] Su H., Wang X., Zhao Q., Jiang C., Zhao Q., Lou W., Wang X. (2026). Polymeric Ionic Liquid–Ti_3_C_2_T_x_ MXene Hybrid Additives for Enhanced Lubrication of Ethylene Glycol/Water Systems. ACS Appl. Mater. Interfaces.

[117] Stoll J.L., Paul M., Pritchett L., Snover A., Woods L., Antony Jose S., Menezes P.L. (2025). Tribology of MXene Materials: Advances, Challenges, and Future Directions. Materials.

[118] Grützmacher P.G., Suarez S., Tolosa A., Gachot C., Song G., Wang B., Presser V., Mücklich F., Anasori B., Rosenkranz A. (2021). Superior Wear-Resistance of Ti_3_C_2_Tx Multilayer Coatings. ACS Nano.

[119] Zhan C., Naguib M., Lukatskaya M., Kent P.R.C., Gogotsi Y., Jiang D. (2018). Understanding the MXene Pseudocapacitance. J. Phys. Chem. Lett..

[120] Chunlim H., Depijan M., Srisawad K., Meekati T., Wongratanaphisan D., Ruankham P., Kanjanaboos P., Pakawatpanurut P. (2025). Solution-Processed, Binder-Free Pristine Ti_3_C_2_T_x_ MXene Electrodes Enabled by MAI Passivation for High-Performance, Scalable Perovskite Solar Cells. Appl. Surf. Sci. Adv..

[121] Liu L., Ying G., Wen D., Zhang K., Hu C., Zheng Y., Zhang C., Wang X., Wang C. (2020). Aqueous Solution-Processed MXene (Ti_3_C_2_T_x_) for Non-Hydrophilic Epoxy Resin-Based Composites with Enhanced Mechanical and Physical Properties. Mater. Des..

[122] Jadhav A., Jha P.K., Salomäki M., Granroth S., Damlin P., Kvarnström C. (2024). Supercapacitive Performance of Ionic-Liquid-Intercalated Two-Dimensional Ti_3_C_2_T_x_ in Redox Electrolyte. Cell Rep. Phys. Sci..

[123] Wang Y., Wang X., Li X., Bai Y., Xiao H., Liu Y., Liu R., Yuan G. (2019). Engineering 3D Ion Transport Channels for Flexible MXene Films with Superior Capacitive Performance. Adv. Funct. Mater..

[124] Bian X., Yang Z., Zhang T., Yu J., Xu G., Chen A., He Q., Pan J. (2023). Multifunctional Flexible AgNW/MXene/PDMS Composite Films for Efficient Electromagnetic Interference Shielding and Strain Sensing. ACS Appl. Mater. Interfaces.

[125] Li Y., Zhang D., Zhou B., He C., Wang B., Feng Y., Liu C. (2022). Synergistically Enhancing Electromagnetic Interference Shielding Performance and Thermal Conductivity of Polyvinylidene Fluoride-Based Lamellar Film with MXene and Graphene. Compos. Part A Appl. Sci. Manuf..

[126] Xia X., Xiao Q. (2021). Electromagnetic Interference Shielding of 2D Transition Metal Carbide (MXene)/Metal Ion Composites. Nanomaterials.

[127] Wang T., Cao H., Ma X., Shen X., Min Y., Xu Q. (2024). Electrodeposited Ti_3_C_2_T_x_ MXene Composite Coating toward Superior Surface Protection on Aluminum Alloy in PEMFC Environments. Corros. Sci..

[128] Thebo K.H., Qian X., Zhang Q., Chen L., Cheng H.-M., Ren W. (2018). Highly Stable Graphene-Oxide-Based Membranes with Superior Permeability. Nat. Commun..

[129] Liu M., Zhuo Y., Sarycheva A., Gogotsi Y., Bissett M.A., Young R.J., Kinloch I.A. (2022). Deformation of and Interfacial Stress Transfer in Ti_3_C_2_ MXene–Polymer Composites. ACS Appl. Mater. Interfaces.

[130] Liang L., Sun X., Ning Y., Wang S., Yin W., Li Y. (2023). Mxene-Toughened Al_2_O_3_ Ceramic at High Temperature. Compos. Part A Appl. Sci. Manuf..

[131] Liu S., Wang S., Sang M., Zhou J., Zhang J., Xuan S., Gong X. (2022). Nacre-Mimetic Hierarchical Architecture in Polyborosiloxane Composites for Synergistically Enhanced Impact Resistance and Ultra-Efficient Electromagnetic Interference Shielding. ACS Nano.

[132] Giménez R., Serrano B., San-Miguel V., Cabanelas J.C. (2022). Recent Advances in MXene/Epoxy Composites: Trends and Prospects. Polymers.

[133] Liu L., Ying G., Sun C., Min H., Zhang J., Zhao Y., Wen D., Ji Z., Liu X., Zhang C. (2021). MXene (Ti_3_C_2_T_x_) Functionalized Short Carbon Fibers as a Cross-Scale Mechanical Reinforcement for Epoxy Composites. Polymers.

[134] Dutta T., Alam P., Mishra S.K. (2025). MXenes and MXene-Based Composites for Biomedical Applications. J. Mater. Chem. B.

[135] Cai Z., Ma Y., Yun M., Wang M., Tong Z., Suhr J., Xiao L., Jia S., Chen X. (2023). Multifunctional MXene/Holey Graphene Films for Electromagnetic Interference Shielding, Joule Heating, and Photothermal Conversion. Compos. Part B Eng..

[136] Wang Y., Yue Y., Cheng F., Cheng Y., Ge B., Liu N., Gao Y. (2022). Ti_3_C_2_T_x_ MXene-Based Flexible Piezoresistive Physical Sensors. ACS Nano.

[137] Venkateshalu S., Grace A.N. (2020). MXenes—A New Class of 2D Layered Materials: Synthesis, Properties, Applications as Supercapacitor Electrode and Beyond. Appl. Mater. Today.

[138] Fei M., Lin R., Lu Y., Zhang X., Bian R., Cheng J., Luo P., Xu C., Cai D. (2017). MXene-Reinforced Alumina Ceramic Composites. Ceram. Int..

[139] Huang Z., Farahmandjou M., Marlton F., Guo X., Gao H., Sun B., Wang G. (2024). Surface and Structure Engineering of MXenes for Rechargeable Batteries beyond Lithium. J. Mater..

[140] Xu S., Wei G., Li J., Ji Y., Klyui N., Izotov V., Han W. (2017). Binder-Free Ti_3_C_2_Tx MXene Electrode Film for Supercapacitor Produced by Electrophoretic Deposition Method. Chem. Eng. J..

[141] Altalbawy F.M.A., Mutar A.A., Jhala R., Patil N., Sead F.F., Shit D., Bupesh Raja V.K., Mahapatro A., Abbas J.K., Noori H. (2025). Advancing Multifunctional Carbon Fibre Composites: The Role of Nanomaterials in Boosting Electrochemical Performance for Energy Storage. R. Soc. Open Sci..

[142] Iqbal A.M., Bhatti H.S. (2025). Current Progress in MXenes for Lithium-Ion Batteries: Critical Role of Interlayer Spacing, Surface Terminal Groups, and Porosity. Phys. Status Solidi (RRL) Rapid Res. Lett..

[143] Shi R., Zhang M., Yan X., Pan J., Moradian J.M., Shahnavaz Z. (2024). Flexible, Self-Supporting Ti_3_C_2_T_x_ Thin Film Electrodes Prepared through Vacuum Filtration and Applied for Supercapacitors. Vacuum.

[144] Yao Y., Li X., Sisican K.M., Ramos R.M.C., Judicpa M., Qin S., Zhang J., Yao J., Razal J.M., Usman K.A.S. (2025). Progress towards Efficient MXene Sensors. Commun. Mater..

[145] Hadi Z., Yeganeh J.K., Zare Y., Naqvi M., Rhee K.Y., Park S.-J. (2025). Analysis of Tunneling Conductivity for MXene Polymer System by the Network of Interphase: Parametric Examinations and Experimental Validation. Sci. Rep..

[146] Monastyreckis G., Stepura A., Soyka Y., Maltanava H., Poznyak S.K., Omastová M., Aniskevich A., Zeleniakiene D. (2021). Strain Sensing Coatings for Large Composite Structures Based on 2D MXene Nanoparticles. Sensors.

[147] Li S., Wu Z., Fan H., Zhong M., Xing X., Wang Y., Yang H., Liu Q., Zhang D. (2025). Flexible Stretchable Strain Sensor Based on LIG/PDMS for Real-Time Health Monitoring of Test Pilots. Sensors.

[148] Hajian S., Maddipatla D., Narakathu B., Atashbar M. (2022). MXene-Based Flexible Sensors: A Review. Front. Sens..

[149] Kong X., Fan C., Liao K., Zhang W., Xiong H. (2025). MXenes for Wearable Pressure Sensing: Progress and Prospects in Human Motion Detection. Alex. Eng. J..

[150] Zheng X., Zhang S., Zhou M., Lu H., Guo S., Zhang Y., Li C., Tan S.C. (2023). MXene Functionalized, Highly Breathable and Sensitive Pressure Sensors with Multi-Layered Porous Structure. Adv. Funct. Mater..

[151] Zhou A., Yi W., Wu Y., Wu Z., Fu Y., Liu T., Li H., Bian N., Liu S. (2025). MXene Aerogel Pressure Sensor Improved by Introducing Intermolecular Forces for Human Motion Detection and Voice Recognition. Adv. Electron. Mater..

[152] Li Z., He Q., Chen H., Chen Y., Zeng C., Xu T., Deng S., Zhang C. (2025). Multifunctional MXene Inks for Printed Electrochemical Energy Storage Devices. Energy Mater..

[153] Cao L.C.T., Hakim L., Nagao Y., Boonruang S., Tseng S.-F., Hsu S.-H. (2025). Self-Powered Humidity Sensor Based on Porous Ti_3_C_2_T_x_ MXene: High Sensitivity and Fast Response for Real-World Applications. Chem. Eng. J..

[154] He T., Liu W., Lv T., Ma M., Liu Z., Vasiliev A., Li X. (2021). MXene/SnO2 Heterojunction Based Chemical Gas Sensors. Sens. Actuators B Chem..

[155] Kim S.J., Koh H.-J., Ren C.E., Kwon O., Maleski K., Cho S.-Y., Anasori B., Kim C.-K., Choi Y.-K., Kim J. (2018). Metallic Ti_3_C_2_T_x_ MXene Gas Sensors with Ultrahigh Signal-to-Noise Ratio. ACS Nano.

[156] Yang J., Dai Q., Wu H., Yang L., Guo S., Zhao Q., Hou M., Komarneni S., Xia Y. (2025). Development of Long-Term Stable MXene-Based Gas Sensing Material. Molecules.

[157] Zhang T., Mazzio K.A., Wang R.J., Lounasvuori M., Al-Temimy A., Amargianou F., Mawass M.-A., Kronast F., Többens D.M., Lips K. (2025). Conductivity Hysteresis in MXene Driven by Structural Dynamics of Nanoconfined Water. Nat. Commun..

[158] Zhao L., Fu X., Xu H., Zheng Y., Han W., Wang L. (2022). Tissue-Like Sodium Alginate-Coated 2D MXene-Based Flexible Temperature Sensors for Full-Range Temperature Monitoring. Adv. Mater. Technol..

[159] Yang W., Liu S., Wang Y., Liu H., Liu C., Shen C. (2024). Advances in Multifunctional Flexible MXene-Based Stress Sensors. J. Mater. Chem. C.

[160] Abdullah A.M., Biswas M.A.S., Dutta A., Li J., Das S., Zhang X., Zhang W., Zohra F.T., Moreno Calva A., Gray J.L. (2025). In Situ Functionalized MXene on Porous Laser-Induced Graphene for Adsorption-Dominated Miniaturized Multifunctional Sensors. ACS Nano.

[161] Wu L., Liu J., Du F., Xia H., Liu P., Luo J., Yang Y. (2025). Bionic Learning in MXene-Based Actuators: An Emerging Frontier. Adv. Colloid Interface Sci..

[162] Wang X., Xue P., Ma S., Gong Y., Xu X. (2023). Polydopamine-Modified MXene-Integrated Poly(N-Isopropylacrylamide) to Construct Ultrafast Photoresponsive Bilayer Hydrogel Actuators with Smart Adhesion. ACS Appl. Mater. Interfaces.

[163] Feng S., Xu G., Deng P., Guo Q., Yi Y., Chen B., Gu Z., Sporrer L., Laval-Schmidt P.-A., Lu C. (2026). Screen-Printed Multifunctional Anti-Counterfeiting MXene-Based Device with Ultra-Fast On-Demand Degradability. Adv. Funct. Mater..

[164] Liu W., Cheng Y., Liu N., Yue Y., Lei D., Su T., Zhu M., Zhang Z., Zeng W., Guo H. (2021). Bionic MXene Actuator with Multiresponsive Modes. Chem. Eng. J..

[165] Shahsa H., Cuvin P., Gu J., Filleter T., Duduta M. (2025). AgNW/Graphene Hybrid Electrode for Dielectric Elastomer Actuator. ACS Omega.

[166] Wyatt B.C., Anasori B. (2022). Self-Assembly and in-Situ Characterization of Ti_3_C_2_T_x_ in Al: A Step toward Additive Manufacturing of MXene-Metal Composites. Appl. Mater. Today.

[167] Chen T., Liu L., Han L., Yu X., Tang X., Li W., Qian Z., Li J., Gan G. (2024). Ultrahigh Thermal Conductivity of Epoxy/Ag Flakes/MXene@Ag Composites Achieved by In Situ Sintering of Silver Nanoparticles. Langmuir.

[168] Koutsioukis A., Ruan S., Cabello R., Lee H., Oikonomou I.Μ., Guo X., Sasnauskas A., Munuera J.M., Rafferty A., Xiong Y. (2026). Sustainable, Solvent-Free Exfoliation of 2D Materials for Thermally Conductive Metal Powder Coatings. npj 2D Mater. Appl..

[169] Mohtasim M.S., Das B.K. (2025). MXene Based Composite Phase Change Materials for Thermal Energy Storage Applications: Featuring Bio-Mimic Approaches. Renew. Sustain. Energy Rev..

[170] Anand S., Vu M.C., Mani D., Kim J.-B., Jeong T.-H., Choi W.-K., Won J.-C., Kim S.-R. (2024). A Continuous Interfacial Bridging Approach to Fabricate Ultrastrong Hydroxylated Carbon Nanotubes Intercalated MXene Films with Superior Electromagnetic Interference Shielding and Thermal Dissipating Properties. Adv. Compos. Hybrid Mater..

[171] Zhang F., Liu W., Wang S., Shi H., Liu C., Liang L., Pi K. (2021). Engineering MXenes (Ti_3_C_2_T_x_) Surface with TiO_2_ for Enhancing Anti-Corrosion Performance of Coatings. Polymer.

[172] Sun W., Yang Y., Yang Z., Wang L., Wang J., Xu D., Liu G. (2021). Review on the Corrosion-Promotion Activity of Graphene and Its Inhibition. J. Mater. Sci. Technol..

[173] Xu R., Kang Y., Zhang W., Pan B., Zhang X. (2023). Two-Dimensional MXene Membranes with Biomimetic Sub-Nanochannels for Enhanced Cation Sieving. Nat. Commun..

[174] Barbhuiya N.H., Singh S.P. (2024). Ex Situ and In Situ Organic Fouling Monitoring in Laser-Induced Graphene-Based Electroconductive Membranes for Desalination and Wastewater Treatment. ACS Appl. Nano Mater..

[175] Sunkara S.V., Manna S., Zambrano D.F., Wyatt B.C., Patenaude J., Wright B.G., Liu Y., Sankaranarayanan S., Anasori B., Rosenkranz A. (2025). Solid Lubricant Molybdenum Based MXene with Prolonged Macroscale Superlubricity. Mater. Today.

[176] Kouchi F.R., Varghese T.V., Burgoyne H., Mansoor N.E., Seol M.-L., McKibben N., Nirantar S., Chinnathambi K., Eixenberger J., Maryon O. (2025). StableTi_3_C_2_T_x_ MXene Ink Formulation and High-Resolution Aerosol Jet Printing for High-Performance MXene Supercapacitors. Small Methods.

[177] Guo Q., Yuan Y., Xu L., Wang W. (2025). Recent Advances in MXene-Based Flame Retardants for Enhancing Fire Safety in Thermoplastic Resins. Fire.

[178] Soomro R.A., Zhang P., Fan B., Wei Y., Xu B. (2023). Progression in the Oxidation Stability of MXenes. Nano-Micro Lett..

[179] Huang W., Zhang Y., Song M., Wang B., Hou H., Hu X., Chen X., Zhai T. (2022). Encapsulation Strategies on 2D Materials for Field Effect Transistors and Photodetectors. Chin. Chem. Lett..

[180] Zhao X., Vashisth A., Prehn E., Sun W., Shah S.A., Habib T., Chen Y., Tan Z., Lutkenhaus J.L., Radovic M. (2019). Antioxidants Unlock Shelf-Stable Ti_3_C_2_T_x_ (MXene) Nanosheet Dispersions. Matter.

[181] Luo X., Wu Y., Hu H., Wei T., Wu B., Ding J., Liu Q., Luo J., Liu X. (2024). Boron-Doped Ti_3_C_2_T_x_ MXene for Effective and Durable High-Current-Density Ammonia Synthesis. Small.

[182] Das S., Shamim S.U.D., Hossain M.K., Ahmed F., Hossain M.A., Rahman M.O. (2022). A Novel Silicon-Doped 2D Ti2C MXene Monolayer as High Capacity Stable Anode Material for Lithium Ion Batteries: Insight from Density Functional Theory Study. Appl. Surf. Sci..

[183] Haleem A., Ullah M., Kiran L., Fan W., Pan J., Li H. (2025). Recent Advancements and Assessments in MXene-Based Composites Stability for Efficient Solar-Heating Water Evaporation: A Systematic and Comprehensive Review. Green Carbon.

[184] Zhang H.-Y., Xiao H., Long J.-J. (2025). Preparation, Structure, Property and Application of MXene in Fabricating Functional and Intelligent Textiles: A Comprehensive Review. Compos. Part B Eng..

[185] Cui J., Wu J., Feng A., Yu Y., Mi L., Yu Y. (2024). Low Infrared Emissivity and Oxidation Stability of Ti_3_C_2_T_x_ MXene-Based Composite with Tannic Acid. Chem. Eng. J..

[186] Zaghdoudi M., Kömmling A., Jaunich M., Wolff D. (2020). Erroneous or Arrhenius: A Degradation Rate-Based Model for EPDM during Homogeneous Ageing. Polymers.

[187] Nouseen S., Pumera M. (2025). Electrochemical Etching of MXenes: Mechanism, Challenges and Future Outlooks. J. Mater. Chem. A.

[188] Kahkesh H., Yeganeh M. (2026). Green Strategies for MXene Synthesis: Toward Sustainable Nanomaterials and Emerging Applications. Results Eng..

[189] Clark M.J., Oakley A.E., Zhelev N., Carravetta M., Byrne T., Nightingale A.M., Bimbo N. (2025). MXene Synthesis in a Semi-Continuous 3D-Printed PVDF Flow Reactor. Nanoscale Adv..

[190] Deng S., Guo T., Nüesch F., Heier J., Zhang C.J. (2023). Stable MXene Dough with Ultrahigh Solid Fraction and Excellent Redispersibility toward Efficient Solution Processing and Industrialization. Adv. Sci..

[191] Jolly S., Paranthaman M.P., Naguib M. (2021). Synthesis of Ti_3_C_2_T_z_ MXene from Low-Cost and Environmentally Friendly Precursors. Mater. Today Adv..

[192] Zha X.-H., Zhou J., Eklund P., Bai X., Du S., Huang Q. (2019). Non-MAX Phase Precursors for MXenes. 2D Metal Carbides and Nitrides (MXenes).

[193] Carey M.S., Barsoum M.W. (2022). Scalable and Sustainable Production of Ti_3_C_2_T*_z_* MXene and Fluorine Recovery from Wastewater through Cryolite Precipitation. RSC Adv..

[194] Riazi H., Anayee M., Hantanasirisakul K., Shamsabadi A.A., Anasori B., Gogotsi Y., Soroush M. (2020). Surface Modification of a MXene by an Aminosilane Coupling Agent. Adv. Mater. Interfaces.

[195] Ko T.Y., Hong J., Lee J., Oh T., Lee A.S., Kim S.J. (2025). Biaryl-Functionalization of MXenes toward Electrically Conductive EMI-Shielding Polymer Composites with Low Percolation Threshold. Chem. Eng. J..

[196] Liu L., Ying G., Jiang Q., Wen D., Wang P., Wu M., Ji Z., Zheng Y., Wang X. (2024). Ultra-High-Temperature Application of MXene: Stabilization of 2D Ti_3_C_2_T_x_ for Cross-Scale Strengthening and Toughening of 3D TiC. J. Adv. Ceram..

[197] Iravani P., Iravani S., Varma R.S. (2022). MXene-Chitosan Composites and Their Biomedical Potentials. Micromachines.

[198] Wu X., Gong J., Zhang H., Wang Y., Tan F. (2024). Cellular Uptake and Cytotoxicity of PEGylated MXene Nanomaterials Mediated by Protein Corona. Sci. Total Environ..

[199] Usman K.A.S., Zhang J., Bi L., Seyedin S., Wang X., Gogotsi Y., Razal J.M. (2025). The Future of MXene Fibers. Adv. Mater..

[200] Firouzjaei M., Nemani K., Sadrzadeh M., Wujcik E., Elliott M., Anasori B. (2023). Life Cycle Assessment of Ti_3_C_2_T_x_ MXene Synthesis. Adv. Mater..

[201] Rahman U.U., Humayun M., Ghani U., Usman M., Ullah H., Khan A., El-Metwaly N.M., Khan A. (2022). MXenes as Emerging Materials: Synthesis, Properties, and Applications. Molecules.

